# Paleobiogeographical origins of *Fasciola hepatica* and *F. gigantica* in light of new DNA sequence characteristics of *F. nyanzae* from hippopotamus

**DOI:** 10.3389/fvets.2022.990872

**Published:** 2022-09-09

**Authors:** María Dolores Bargues, Ali Halajian, Patricio Artigas, Wilmien J. Luus-Powell, M. Adela Valero, Santiago Mas-Coma

**Affiliations:** ^1^Departamento de Parasitologia, Facultad de Farmacia, Universidad de Valencia, Valencia, Spain; ^2^Centro de Investigación Biomédica en Red (CIBER) de Enfermedades Infecciosas, Instituto de Salud Carlos IIII, Madrid, Spain; ^3^DSI-NRF SARChi Chair (Ecosystem Health), Department of Biodiversity, University of Limpopo, Sovenga, South Africa; ^4^Research Administration and Development, University of Limpopo, Sovenga, South Africa

**Keywords:** *Fasciola* and *Fascioloides* species, paleobiogeographical origins, *F. nyanzae* from hippopotamus, lymnaeid snail vectors, southeastern Africa, Asian Near East

## Abstract

Fascioliasis is a highly pathogenic disease affecting humans and livestock worldwide. It is caused by the liver flukes *Fasciola hepatica* transmitted by *Galba*/*Fossaria* lymnaeid snails in Europe, Asia, Africa, the Americas and Oceania, and *F. gigantica* transmitted by *Radix* lymnaeids in Africa and Asia. An evident founder effect appears in genetic studies as the consequence of their spread by human-guided movements of domestic ruminants, equines and Old World camelids in the post-domestication period from the beginning of the Neolithic. Establishing the geographical origins of fasciolid expansion is multidisciplinary crucial for disease assessment. Sequencing of selected nuclear ribosomal and mitochondrial DNA markers of *F. nyanzae* infecting hippopotamuses (*Hippopotamus amphibius*) in South Africa and their comparative analyses with *F. hepatica* and *F. gigantica*, and the two *Fascioloides* species, *Fs. jacksoni* from Asian elephants and *Fs. magna* from Holarctic cervids, allow to draw a tuned-up evolutionary scenario during the pre-domestication period. Close sequence similarities indicate a direct derivation of *F. hepatica* and *F. gigantica* from *F. nyanzae* by speciation after host capture phenomena. Phylogenetic reconstruction, genetic distances and divergence estimates fully fit fossil knowledge, past interconnecting bridges between continents, present fasciolid infection in the wild fauna, and lymnaeid distribution. The paleobiogeographical analyses suggest an origin for *F. gigantica* by transfer from primitive hippopotamuses to grazing bovid ancestors of Reduncinae, Bovinae and Alcelaphinae, by keeping the same vector *Radix natalensis* in warm lowlands of southeastern Africa in the mid-Miocene, around 13.5 mya. The origin of *F. hepatica* should have occurred after capture from primitive, less amphibious *Hexaprotodon* hippopotamuses to mid-sized ovicaprines as the wild bezoar *Capra aegagrus* and the wild mouflon *Ovis gmelini*, and from *R. natalensis* to *Galba truncatula* in cooler areas and mountainous foothills of Asian Near East in the latest Miocene to Early Pliocene, around 6.0 to 4.0 mya and perhaps shortly afterwards.

## Introduction

The trematode species *Fasciola hepatica* and *F. gigantica* are liver flukes which infect livestock and humans causing a worldwide parasitic disease called fascioliasis. These two helminths are transmitted by specific freshwater snails of the family Lymnaeidae, including species of the *Galba*/*Fossaria* group in the transmission of *F. hepatica* in Europe, Asia, Africa, the Americas and Oceania, and species of the *Radix* group as intermediate hosts or vectors of *F. gigantica* in Africa and Asia (1). The zoonotic characteristics of this disease are due to the low specificity of the adult stage, which is able to develop in many different definitive hosts, mainly herbivore mammals and also omnivores such as humans and pigs. However, these liver flukes do not produce eggs in all definitive host species and, if they do, the fasciolid eggs shed by several definitive host species show low viability rates which are insufficient to maintain the parasite life cycle in the long term and therefore they do not play the role of a reservoir host (2).

Ruminants such as sheep, goats, cattle and buffaloes are the main reservoirs. Secondarily, equids and Old World camelids also participate in the transmission and spread of this disease typical of rural areas (1). The high pathogenicity along the first invasive, migratory or acute phase of the disease and during the long biliary, obstructive or chronic phase underlies great veterinary losses and impact on livestock husbandry (3). In public health, human fascioliasis has been emerging as a wide problem along the last three decades (4), with the description of human fascioliasis endemic areas in many countries and a progressively increasing number of case reports in the five continents (5). This worrying scenario adds to its high pathogenicity (6–8), virulence (9), long-term post-treatment sequelae (10), and immunosuppression in the acute phase (11) and chronic phase (12, 13). The latter underlies usual coinfections with other pathogenic protozoans and helminths leading to high morbidity (14, 15), and even mortality, mainly in hyperendemic areas of low income countries (16). According to all this, the World Health Organization (WHO) included fascioliasis among the group of Foodborne Trematodiases in the priority list of Neglected Tropical Diseases (NTDs) in the WHO NTD Roadmap for 2030 (17), and has very recently emphasized the convenience of achieving the Roadmap targets through a cross-cutting One Health approach (18).

This disease is the only trematodiasis showing a worldwide distribution. It reached such a wide coverage by taking advantage of human-guided movements of domestic ruminants, equids and Old World camelids along the 12,000–10,000 year post-domestication period. This was possible due to the wide geographical distribution of their snail vector species belonging to Lymnaeidae, a very old family of snails with species present in all continents. Moreover, the amphibious characteristics of these snails facilitate their passive transport by domestic animals when in mud attached to their hooves. Reproduction by selfing and quick multiplication further add to the high spreading capacity of these snails. Thus, the large animal-accompanied movements of human populations throughout the Neolithic, and also subsequently in present times, were decisive in fasciolid spread. DNA marker sequencing indicated a worldwide radiation with evident founder effect from the main domestication regions, illustrating the link of these parasites to mankind history (1). Similar results have been recently observed in genome-wide comparisons of these trematodes (19).

Therefore, assessing the paleobiogeographical origins of both *F. hepatica* and *F. gigantica* in the pre-domestication period is crucial to establish the sources of their radiations. This enables to understand the spreading capacities of these parasites and offers the baseline for disease control and prevention measures. It should be considered that this human disease follows different transmission patterns and shows different epidemiological situations. This is in part due to the diversity of human infection sources linked to diet, behavior and traditions markedly differing according to human population ethnics (20). However, the aforementioned disease heterogeneity also depends on (i) which *Fasciola* species and the presence of only one or the two species in the same area, (ii) the number of lymnaeid vector species involved and their ecology, behavior, population dynamics and seasonality, and also (iii) the mammal reservoir species participating in the disease transmission. All these factors are partly consequences of previous introductions by human-guided animal movements when the range of environmental and climatic characteristics allows for fasciolids and lymnaeids to colonize and establish in an area according to their development requirement thresholds.

**Figure 1:**
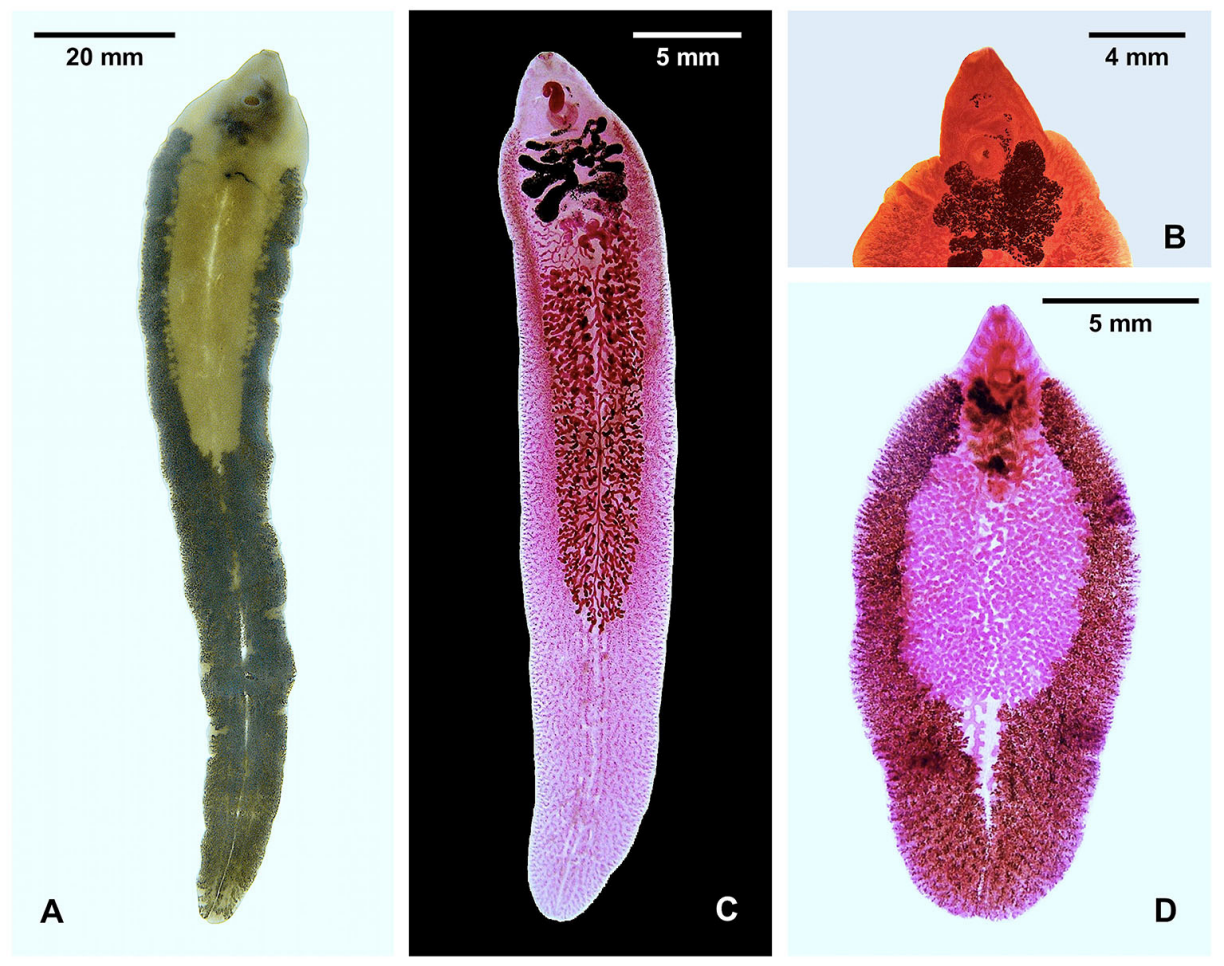
Species of *Fasciola*. **(A)** Unstained *F. nyanzae* from hippopotamus in South Africa (note maximum width at the level of ovary). **(B)** Anterior part of stained *F. nyanzae* showing big apical cone and pronounced shoulders. **(C)** *F. gigantica* from cattle in Burkina Faso (note less pronounced shoulders and almost parallel lateral body walls). **(D)** Specimen highlighting the vitelline follicles of *F. hepatica* from sheep in Spain (note non-parallel lateral body walls). See decreasing distance between testis end and posterior extremity of the body.

The only two other species included in the genus *Fasciola* are *F. nyanzae* infecting hippopotamuses in Africa and *F. jacksoni* specific of Asian elephants (1). However, the mitochondrial genome of the latter has recently demonstrated that this species should better be included in the other fasciolid genus *Fascioloides* (21). *Fascioloides* was so far only including the large American liver fluke species *Fascioloides magna* which infects cervids and bovids originally in North American and recently anthropogenically introduced into Europe (22, 23). The recent finding of *F. nyanzae* infecting hippopotamuses in three natural reserves of northeastern South Africa (Figure 1) has given the chance for the obtaining of the complete sequences of the DNA markers of the 18S gene and the spacers ITS-1 and ITS-2 of the nuclear ribosomal operon (rDNA), and the *cox*1 and *nad*1 genes of the mitochondrial genome (mtDNA).

This trematode material enables not only for the analysis of the intraspecific variability of *F. nyanzae*, but also the comparison of its sequences with those of *F. hepatica* and *F. gigantica* (Figure 1), as well as with those of *Fs. jacksoni* and *Fs. magna*. The purpose of the present study includes genetic distances and divergence estimates by means of the 18S molecular clock assessment, to allow for an accurate re-definition of the palaeobiogeographical origins of *F. hepatica* and *F. gigantica*, by additionally considering the present paleontological knowledge on the evolution of hippopotamuses and ruminants, present data on the fasciolid infection in the wild fauna, and the geographical distribution of the respective lymnaeid vector species.

## Materials and methods

### Sampling for DNA markers and potential drawback analyses

For the characterization of the fasciolid flukes, the sequences of the complete rDNA small subunit (18S rRNA gene), the complete inter-genic nuclear ribosomal DNA (rDNA) region including the spacers ITS-1 and ITS-2 and the 5.8S gene, and the complete sequences of the two protein-coding genes *cox*1 and *nad*1 of the mitochondrial DNA (mtDNA) were selected according to the purposes of this study. The usefulness of these markers for the molecular characterization of *Fasciola* species and strains has already been verified both at local and regional levels (24, 25) and in worldwide analyses, including the assessment of the spreading routes (1).

Only adult flukes collected from livers of the respective hosts were used for the sequences obtained in the present study. All hosts furnishing fasciolid materials were dead before the autopsy. Only fasciolid samples from selected countries (whenever possible personally collected to assure the obtaining of complete information details) and sequenced by the authors are included in this study. This allowed each marker to be sequenced in its complete length and each mutation detected to be verified in the electropherograms and by specimens re-sequencing when needed. Only in the case of *Fs. magna*, sequences were used from other authors available in GenBank. No sequence fragments were used to avoid information bias (26).

Countries for the obtaining of fasciolid species were selected to assure that only “genetically pure” specimens were used for sequencing. Samples of *F. hepatica* were only from countries of Europe and Latin America (see list of countries in next section), where hybrids do not occur because of the absence of *F. gigantica* and *Radix* lymnaeids (25). Similarly, samples of *F. gigantica* were only from African countries (see list of countries in next section), where hybrids do not occur because of the absence of *F. hepatica* and *Galba*/*Fossaria* lymnaeids (1, 27). Specimens of *F. hepatica* and *F. gigantica* were obtained from cattle and sheep. It should be considered that metacercariae of these two fasciolid species are similarly infective to cattle and sheep independently of the host isolate of origin (28).

**Figure 2:**
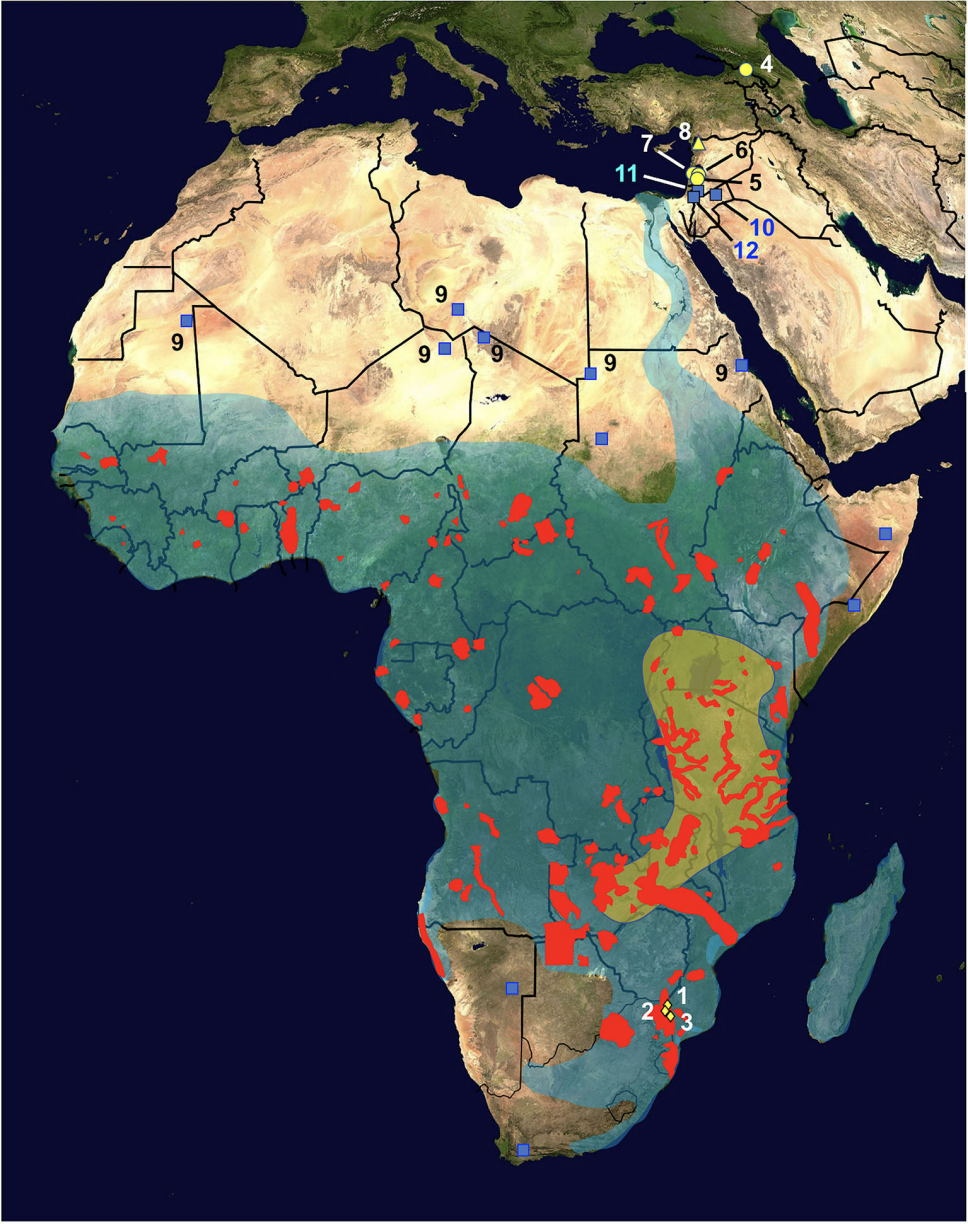
Map of Africa and Asian Near East showing the distribution of hippopotamus definitive host and radicine snail vector of *Fasciola nyanzae*. Diamonds = natural reserves where the infected hippopotamuses were found, located in the Mpumalanga province, northeastern South Africa, including Private Nature Reserve 1, Hoedspruit (No. 1), Private Nature Reserve 3 (New camp dam) (2), and Private Nature Reserve 2 (3); red areas = present fragmented distribution of *Hippopotamus amphibius*; transparent green area = oldest region of the African hippopotamus according to molecular assessments; yellow circles = fossil remains of *Hexaprotodon georgicus* in the Caucasus mountains (4) and *Hexaprotodon gorgops* in the Levantine corridor in archeological sites of northern Israel in Ubeidiyah in the Jordan Rift Valley (5), Gesher Benot Yaakov (6), and Evron (7); yellow triangle = fossil remains of *H. amphibius* found in Latamne, Syria (8); transparent blue area = present distribution of its snail vector *Radix natalensis*; blue squares = isolated populations of *R. natalensis* outside the general area, including fossil ones in the Sahara desert (9); note populations in Jordan found in Azraq oasis (10) and the Jordan Valley (11), and in Kishda, Palestina (12). Information from various sources (see text). Geographic background from composed satellite map of Africa orthographic projection by NASA (public domain) *via* Wikimedia Commons. Schema S. Mas-Coma.

*Fasciola nyanzae* poses no problem in this sense, because it is specific to hippopotamus (29). A total of 15 liver fluke specimens were obtained from naturally infected *Hippopotamus amphibius* (two males and five females, aged from 7 to 15 years old), from the following three nature reserves in South Africa: (i) eight liver fluke specimens from Private Nature Reserve 1, Hoedspruit, (ii) one liver fluke specimen Private Nature Reserve 3 (New camp dam), and (iii) six liver fluke specimens from Nature Reserve 2, all located in the Mpumalanga Province (Figure 2). Unfortunately, we could not extract proper DNA from the only specimen from the Private Nature Reserve 3 because of bad fixation.

*Fascioloides jacksoni* is specific to the Asian elephant and it was collected on the island of Sri Lanka, where both *F. hepatica* and *F. gigantica* are absent. Regarding *Fs. magna*, this is the only species whose sequences were obtained from GenBank and a priori its potential hybridization capacity with *F. hepatica* or *F. gigantica* may be excluded, as such hybrids have never been reported.

In the case of the three lymnaeid vector species considered for the present paleobiogegraphical analysis, namely *Radix natalensis, R. auricularia* and *Galba truncatula*, only the rDNA markers of the 18S gene and the spacers ITS-1 and ITS-2 were obtained and used for the analysis of relative genetic divergences by means of molecular clock assessments.

### Fasciolid and lymnaeid samples

Specimens of the fasciolid species were collected from various countries representing different regions, full-length sequences of the aforementioned rDNA and mtDNA markers obtained and deposited in GenBank (Accession Numbers noted in parentheses), and used for the assessments in this paleobiogeographical study:

**18S rDNA:** 21 sequences including:

- *F. hepatica:* six sequences from Spain (two), Peru (two) and Argentina (two) (ON661086).
- *F. gigantica:* six sequences from Burkina Faso (two), Nigeria (two) and Cameroon (two) (ON661087- ON661089).
- *F. nyanzae*: six sequences from (i) Private Nature Reserve 1, Hoedspruit (three) and (ii) Private Nature Reserve 2 (three), South Africa (ON661084).
- *Fs. jacksoni*: three sequences from Sri Lanka (ON661085).

**Intergenic rDNA region (ITS1-5.8S-ITS2):** 64 sequences including:

- *F. hepatica:* 18 sequences including 10 (one by country) from Spain, France, Poland, Mexico, Venezuela, Ecuador, Peru, Bolivia, Uruguay, and Argentina (MG569980), six (one by country) from Spain, Andorra, Mexico, Ecuador, Bolivia, and Uruguay (MG569978, MG569981), and two from Ecuador (MK212150).
- *F. gigantica:* 30 sequences from Burkina Faso (15), Niger (three), Nigeria (five), Senegal (three), Cameroon (two) (AJ853848), and Algeria (two) (ON661090, ON661091).
- *F. nyanzae*: 13 sequences from Private Nature Reserve 1, Hoedspruit (seven) and Private Nature Reserve 2 (six), South Africa (ON661092).
- *Fs. jacksoni*: three sequences from Sri Lanka (ON661093).

**mtDNA** ***cox*1 gene:** 57 sequences including:

- *F. hepatica*: 10 sequences from Spain, Poland, Mexico, Venezuela, Ecuador, Peru, Bolivia, Uruguay, and Argentina (MW867310- MW867317, MW867324-MW867326).
- *F. gigantica:* 31 sequences from Burkina Faso (15), Niger (three), Nigeria (five), Senegal (three), Cameroon (two) (MT094380-MT094390), and Algeria (three) (MN913872, MN913873).
- *F. nyanzae*: 13 sequences from the Private Nature Reserve 1, Hoedspruit (seven) and the Private Nature Reserve 2 (six), South Africa (ON661094-ON661100).
- *Fs. jacksoni*: three sequences from Sri Lanka (ON733331).

**mtDNA** ***nad*1 gene:** 57 sequences including:

- *F. hepatica*: 10 sequences from Spain, Poland, Mexico, Venezuela, Ecuador, Peru, Bolivia, Uruguay, and Argentina (MW867318-MW867323, MW867327-MW867329).
- *F. gigantica*: 31 sequences from Burkina Faso (15), Niger (three), Nigeria (five), Senegal (three), Cameroon (two) (MT094391-MT094405), and Algeria (three) (MN913874).
- *F. nyanzae*: 13 sequences from Private Nature Reserve 1, Hoedspruit (seven) and the Private Nature Reserve 2 (six), South Africa (ON661077-ON661084).
- *Fs. jacksoni*: three sequences from Sri Lanka (ON713419).

For comparison purposes, the following sequences of *F. nyanzae* and *Fs. magna* were obtained from GenBank. Concerning *F. nyanzae*, sequences retrieved included ITS-1 and ITS-2 (MT909820-21, MW046870, MW046872, MT893586-MT893588, MT893595) and partial *cox*1 sequences (MT909542- MT909550) from Zimbabwe (30). Concerning *Fs. magna*, sequences retrieved included the 18S gene (EF051080) and ITS-1 and ITS-2 (EF051080, KU232369) from USA (31, 32), and *cox*1 (EF534998) and *nad*1 (EF535001) from USA (33). *Fasciolopsis buski* 18S and ITSs (L06668, MN970005) (34, 35) and *cox*1 (MF287794) and *nad*1 (MF287793) from Vietnam (36) were included in the corresponding data matrices for phylogenetic reconstructions.

Additionally, specimens of the three lymnaeid snail vector species involved in the present study were collected in 14 different countries, full-length sequences of the following nuclear rDNA markers obtained and deposited in the GenBank (Accession Numbers noted in parentheses), and used for the assessments of relative genetic divergence estimates:

**18S rDNA:** 18 sequences including:

- *R. natalensis*: six sequences from Egypt (one), Burkina Faso (three), and Angola (two) (ON720979).
- *R. auricularia*: six sequences from Spain (two), France (one), Georgia (two) and Iran (one) (Z73980).
- *G. truncatula*: six sequences from Spain (two), Egypt (one), Georgia (two), and Iran (one) (Z73985).

**rDNA ITS-2:** 41 sequences including:

- *R. natalensis*: 13 sequences from Egypt (two), Burkina Faso (six), Nigeria (one) and Angola (four) (ON729287, ON729288).
- *R. auricularia*: 14 sequences from Spain (three), France (three), Czech Republic (two), Georgia (four) and Iran (two) (AJ319628-AJ319632, ON729289, ON729290).
- *G. truncatula*: 14 sequences representing Spain, France, Morocco, Egypt, Georgia, Iran Venezuela, Bolivia, and Argentina (AJ243017, AJ296271, AJ272051).

### DNA sequencing

For DNA extraction, a small part of the anterior body region of fasciolids was individually processed (24, 25, 37). Materials were suspended in 400 μl of lysis buffer (10 mM Tris-HCl, pH 8.0, 100 mM EDTA, 100 mM NaCl, 1% sodium dodecyl sulfate SDS) containing 500 μg/ml Proteinase K (Promega, Madison, WI, USA). The digestion was performed during 2 h at 55°C, including alternate shaking every 15 min. Methods previously outlined were followed concerning the procedure steps (24, 38). The phenol-chloroform extraction and ethanol precipitation method was applied for total DNA isolation. Each pellet was dried and resuspended in 30 μl sterile TE buffer (pH 8.0), and subsequently this suspension was stored at −20°C until needed.

Each DNA marker was amplified by PCR in an independent way for each liver fluke individual. Each PCR product was sequenced for a bona-fide haplotype characterization. A set of eight conserved oligonucleotide primers was used for the amplification of five superimposed fragments of the 18S rRNA gene using specific primers (26, 38, 39) and a standard protocol to amplify specific 18S rDNA regions (39). Forward and reverse primers were designed in the regions flanking the rRNA genes 18S and 28S for the subsequent amplification of the complete ITS-1, 5.8S, ITS-2 region (24, 37). The complete *cox*1 and *nad*1 gene sequences were obtained using forward and reverse primers designed in regions flanking these genes (1, 24, 27).

For the PCR amplification, the Biotools DNA polymerase® (Biotools B&M Labs. S.A., Madrid, Spain) was used in a Verity-96 Well Thermal Cycler (Applied Biosystems, Thermo Fisher Scientific, Waltham, MA USA). The programs differed according to the marker.

– For the 18S rDNA, it comprised one cycle of 3 min at 94°C, 34–39 cycles of 30 min at 94°C, 40 s at 50–53°C and 1.5 min at 72°C each, preceded by 3 min at 72°C, and followed by a final cooling at 4°C.
– For the rDNA inter-genic region, it comprised one cycle of 2 min at 94°C, 35 cycles of 1 min at 93°C, 1 min at 55°C and 1 min at 72°C each, preceded by 2 min at 72°C, and followed by a final cooling at 4°C.
– For the mtDNA *cox*1 and *nad*1 genes, it comprised one cycle of 1 min at 94°C, 40–42 cycles of 1 min at 93°C, 1 min at 52–55°C and 2–3 min at 72°C each, preceded by 5 min at 72°C and followed by a final cooling at 4°C.

For the purification of the PCR product, the Ultra Clean™ PCR Clean-up DNA Purification System (MoBio, Solana Beach, CA, USA) was used following the manufacturer's protocol and eluted in 50 μl of 10 mM TE buffer (pH 7.6). The final DNA concentration (in μg/ml) and the absorbance at 260/280 nm were determined in a Eppendorf BioPhotometer (Hamburg, Germany).

Each molecular marker was sequenced on both strands by the dideoxy chain-termination method performed with the Taq dye-terminator chemistry kit on an Applied Biosystems 3730xl DNA Analyzer (Applied Biosystems, Foster City, CA, USA), by using the PCR primers.

### Sequence analyses

The software Sequencher v. 5.4.6 (Gene Codes Co. MI, USA) was used to edit and assemble the sequences, and ClustalW to align them by means of default parameters in MEGA X software (40). Corresponding penalties for gaps were included in pairwise and multiple alignments. Total character differences were used to measure the divergence of the sequences within and among each one of the 18S, ITS-1 and ITS-2, *cox*1 and *nad*1 markers. All changes, comprising transitions (ts), transversions (tv) and insertions/deletions (indels), were considered as character states in MEGA X.

By means of the ALTER web server (41), the sequences aligned were collapsed to haplotypes, counting gaps as differences. Closely related sequences were searched by utilizing the BLASTN programme from the National Center for Biotechnology Information website (http://www.ncbi.nlm.nih.gov/BLAST). The sequences were analyzed by comparing the rDNA and mtDNA sequences of *F. hepatica, F. gigantica* and *F. nyanzae* in the genus *Fasciola*, and those of *Fs. jacksoni* together with GenBank sequences of *Fs. magna* in the genus *Fascioloides*.

### DNA haplotype nomenclature

The terminology to identify the haplotype (H) of the aforementioned DNA markers follows the previously proposed combined haplotyping (CH) nomenclature (1, 42). According to this nomenclature, 18S haplotypes are defined by numbers, ITS-2 haplotypes are defined by numbers, and ITS-1 haplotypes by capital letters. Numbers are also utilized for the nucleotide and protein haplotypes of the mtDNA *cox*1 and *nad*1 genes. Worth mentioning is that haplotype codes are only definitive when the sequences are complete, i.e., full length sequences. When dealing with fragments or incomplete sequences, haplotype codes are considered only provisional.

### Phylogenetic analyses

Due to different limitations recently shown by mtDNA markers for interspecific sequence analyses in invertebrates (26, 43, 44), phylogenetic reconstruction by combined sequences data sets was made from ribosomal and mitochondrial markers separately. Three different phylogenetic analyses were performed, including only complete sequences of the respective markers.

A first phylogenetic tree was obtained by using the concatenated rDNA sequences of the 18S gene, the ITS-1, the 5.8S gene and the ITS-2. The data matrix included 12 sequences and 3,092 characters. In this analysis, the trematode species *Paramphistomum cervi* (KJ459937) was included as outgroup. Sequences of the Giant Asian fasciolid species *Fasciolopsis buski* infecting the pig and humans in the Far East and southern Asia (45) were also included (L06668 and MN970005). It should be here considered that sequences of nuclear rDNA and mtDNA markers have recently suggested that *Fps. buski* populations from China and Vietnam are similar whereas those of India show sufficient nucleotide differences as to consider that they represent distinct taxa (46).

The second phylogenetic tree was obtained by using the mtDNA *cox*1 sequences, with the data matrix including 33 sequences and 1,578 characters and using *Fps. buski* from Vietnam (MF287794) as outgroup. A third phylogenetic tree was obtained by means of the mtDNA *nad*1 sequences, with the corresponding data matrix including 33 sequences and 903 characters and using *Fps. buski* from Vietnam (MF287793) as outgroup. In both analyses, haplotypes of *F. hepatica, F. gigantica F. nyanzae, Fs. jacksoni* and *Fs. magna* were included.

For the phylogenetic reconstruction in each of the aforementioned trees, all the alignments were visually inspected for anomalies and trimmed to the largest possible consensus. The best substitution model selection analysis was run in MEGA X, considering the BIC scores (Bayesian Information Criterion), the AICc value (Akaike Information Criterion, corrected), the Maximum Likelihood value (lnL) and the number of parameters (including branch lengths) for each model. The evolutionary history was inferred by using the Maximum Likelihood (ML) and the Neighbor-Joining (NJ) methods. Initial tree(s) for the heuristic search were obtained automatically with the Nearest-Neighbor-Interchange (NNI) method by applying Neighbor-Join and BioNJ algorithms to a matrix of pairwise distances estimated using the Maximum Composite Likelihood (MCL) approach. To assess the reliability of the nodes in the ML and NJ trees a bootstrap analysis using 1,000 replicates was made using Bootstrap method in MEGA X.

## Results

### Nuclear rDNA sequences

The small subunit or 18S gene of *F. nyanzae* has a length of 1,980 bp and a GC content of 50.91%. The sequence of this gene proved to be identical in specimens of *F. nyanzae* collected in hippopotamuses inhabiting the Klaserie Private Nature Reserve, Hoedspruit and the Sabi Sand Nature Reserve, located in the Mpumalanga Province (Figure 2). For the analysis of this sequence, it was aligned with those of the same gene we obtained in the close fasciolid species *F. gigantica, F. hepatica* and *Fs. jacksoni* from the elephant in Sri Lanka, plus that of *Fs. magna* infecting cervids and bovids obtained in the USA. The resulting alignment was 1,980-bp long, including 1,966 conserved and 14 variable positions, of which eight were parsimony informative positions and six were singleton sites (Table 1).

**Table 1:** Polymorphic sites in the sequence comparison of the complete 18S rRNA gene sequence of *Fasciola hepatica, F. gigantica* and *F. nyanzae* together with *Fascioloides jacksoni* and *Fs. magna*.

**Fasciolid species**	**18S haplotypes**	**Country**	**GenBank Acc. No**.	**18S rRNA gene variable positions**				**Length (bp)**	**GC (%)**
						11	11		
					1	3377777846	89		
				1	1111111178	1501999299	70		
				1234567890	1234567818	2152034917	71		
*F. hepatica*	Fh-18S-1	Spain	ON661086	CTGGTTGATC	CTGCCAGTTG	TCGTTCTTTT	TT	1,980	50.91
*F. hepatica*	Fh-18S-1	Peru	ON661086	. . . . . . . . . .	. . . . . . . . . G	. . . . . . . . . .	. .	1,980	50.91
*F. hepatica*	Fh-18S-1	Argentina	ON661086	. . . . . . . . . .	. . . . . . . . . G	. . . . . . . . . .	. .	1,980	50.91
*F. gigantica*	Fg-18S-1	Burkina Faso	ON661089	. . . . . . . . . .	. . . . . . . . GG	. . . . . . C . . .	C.	1,980	51.06
*F. gigantica*	Fg-18S-1	Nigeria	ON661088	. . . . . . . . . .	. . . . . . . . GG	. . . . . . C . . .	C.	1,980	51.06
*F. gigantica*	Fg-18S-1	Cameroon	ON661087	. . . . . . . . . .	. . . . . . . . GG	. . . . . . C . . .	C.	1,980	51.06
*F. nyanzae*	Fn-18S-1	South Africa-K	ON661084	. . . . . . . . . .	. . . . . . . . . G	. . . . . . . . . .	. .	1,980	50.91
*F. nyanzae*	Fn-18S-1	South Africa-S	ON661084	. . . . . . . . . .	. . . . . . . . . G	. . . . . . . . . .	. .	1,980	50.91
*Fs. jacksoni*	Fsj-18S-1	Sri Lanka	ON661085	. . . . . . . . . .	. . . . . . . . . A	. . . CCT . ACC	. C	1,980	51.06
*Fs. magna*	Fsm-18S-1	Oregon, USA	EF051080	- - - - - - - - - -	- - - - - - - -. A	CTAC . TCA . .	. C	1,962	50.87

Positions, numbers (to be read in vertical) refer to variable positions obtained in the alignment made with MEGA X; . = identical; shaded positions with a gap = not sequenced; K, Klaserie Private Nature Reserve; S, Sabi Sand Nature Reserve.

This sequence is identical to that of the 18S gene in *F. hepatica*, but shows three mutations with regard to *F. gigantica*, despite identical length, in positions 71 (T in *F. nyanzae* but G in *F. gigantica*), 794 (T vs. C), and 1,878 (T vs. C) (Table 1). This gene is also 1,980-bp long in *Fs. jacksoni*, but has been reported to only have 1,962 bp (lacking 18 nucleotides in the 5' extremity) in *Fs. magna*. When comparing in the corresponding alignment (Table 1), eight mutations appear between *F. nyanzae* and *Fs. jacksoni*, all of them in positions differing from the aforementioned three ones distinaguishing *F. nyanzae* from *F. gigantica*. Nine mutations appear between *F. nyanzae* and *Fs. magna*, of which seven are different from the eight in *Fs. jacksoni*.

The whole intergenic region of the rDNA operon of *F. nyanzae*, including ITS-1, 5.8S gene and ITS-2, proved to be 951-bp long, with a 50.47% GC content. No intraspecific variability was found in the specimens from the two reserves. The lengths of these three markers were 432, 154, and 365 bp, respectively. These lengths agree with the 951-bp-long intergenic region of *F. hepatica* and differ from the 950-bp-long sequence of *F. gigantica* because of one deletion in the ITS-2 of this species. The alignment of the intergenic sequences of these three *Fasciola* species allows for the comparison of the two spacer markers (Table 2). Interestingly, nucleotides differing between the ITS sequences of *F. nyanzae* and those of *F. hepatica* and *F. gigantica* are very few and only appear in the same positions differing between the two latter. Moreover, the differing nucleotides in *F. nyanzae* coincide with those of one or the other of the two fasciolids, which indicates that the ITSs of *F. nyanzae* are of intermediate characteristics. Without counting the two positions of the ITS-2 in which intraspecific variability is known in *F. hepatica* and *F. gigantica, F. nyanzae* only showed two differences in the ITS-1 and other two in the ITS-2 when compared to *F. hepatica*, whereas three and two, respectively appeared when compared to *F. gigantica*.

**Table 2:** Polymorphic sites in the sequence comparison of the nuclear rDNA complete intergenic region and in the ITS-1 and ITS-2 between haplotypes of “genetically pure” *Fasciola hepatica* from livestock in Europe and the Americas, haplotypes of “genetically pure” *F. gigantica* from livestock in African countries, and the haplotype of *F. nyanzae* from hippopotamuses in the nature reserves in South Africa.

***Fasciola* spp. and combined haplotypes**	**Polymorphic sites** **Intergenic region (ITS-1, 5.8S, ITS-2)**											
**Positions**	**24**	**114**	**208**	**286**	**306**	**821**	**834**	**860**	**866**	**874**	**917**	**924**
	**Polymorphic sites ITS-1**					**Polymorphic sites ITS-2**						
**Positions**	**24**	**114**	**208**	**286**	**306**	**234**	**248**	**273**	**279**	**288**	**330**	**337**
*F. hepatica* 1A	C	A	C	T	C	T	A	C	C	C	T	G
*F. hepatica* 2A	C	A	C	T	C	T	A	C	C	T	T	G
*F. hepatica* 1/2A^*^	C	A	C	T	C	T	A	C	C	C/T	T	G
*F. gigantica* 1A	T	T	T	A	T	C	A	T	T	C	-	A
*F. gigantica* 2A	T	T	T	A	T	C	C	T	T	C	-	A
*F. gigantica* 1/2A^**^	T	T	T	A	T	C	C/A	T	T	C	-	A
*F. nyanzae* 1A	T	A	T	T	C	T	A	T	C	C	T	A

* Heterozygotic in position 874/288 not differentiating between *F. hepatica* and *F. gigantica*.

** Heterozygotic in position 834/248 not differentiating between *F. hepatica* and *F. gigantica*- also designed as H3A in Chougar et al. (27).

Opposite to this, nucleotide differences were numerous in the two spacers when compared to *Fs. jacksoni* and *Fs. magna* (Tables 3, 4) and underlie the great genetic distances between the two genera *Fasciola* and *Fascioloides* (Tables 5A–C), which agree with the marked morpho-anatomical differences in the adult stages of their species.

**Table 3:** Polymorphic sites in the sequence comparison of the nuclear rDNA ITS-1 between haplotypes of “genetically pure” *Fasciola hepatica* from livestock in Europe and the Americas, “genetically pure” *F. gigantica* from livestock in African countries, *F. nyanzae* from hippopotamuses in the nature reserves in South Africa and from snails and one hippopotamus in Zimbabwe, *Fascioloides jacksoni* from elephants in Sri Lanka, and *Fs. magna* from cervids in USA.

**Fasciolid species**	**haplotype or isolate (I)**	**Country**	**Acc. No**.	**ITS-1 variable positions**				**Length (bp)**	**GC (%)**
					111111	1111111222	2222333333		
				1244577777	7799111246	6677789056	7789001227		
				6568902345	6737356275	6826735977	2878571591		
*F. hepatica*	Fh-ITS1-A	Spain + a	MG569980	-CACGTGCGG	ACAAGACTCT	CCGTTAGCTT	T A T A T C A T C C	365	48.49
*F. gigantica*	Fg-ITS1-A	Burkina Faso + b	AJ853848	-T . . . . . . . .	. . . . . T . . . .	. . . . . . . T . .	. . A . . T . . . .	364	48.08
*F. nyanzae*	Fn-ITS1-A	South Africa-K	ON661092	-T . . . . . . . .	. . . . . . . . . .	. . . . . . . T . .	. . . . . . . . . .	365	47.95
*F. nyanzae*	Fn-ITS1-A	South Africa-S	ON661092	-T . . . . . . . .	. . . . . . . . . .	. . . . . . . T . .	. . . . . . . . . .	365	47.95
*F. nyanzae*	I S8bLN	Zimbabwe	MT893595	-T . . . . . . . .	. . . . . . . . . .	. . . . . . . T . .	. . . . . . . . . .	365	47.95
*F. nyanzae*	I 483	Zimbabwe	MT893588	-T . . . . . . . .	. . . . . . . . . .	. . . . . . . T . .	. . . . . . . . . .	365	47.95
*F. nyanzae*	I 481	Zimbabwe	MT893587	-T . . . . . . . .	. . . . . . . . . .	. . . . . . . T . .	. . . . . . . . . .	365	47.95
*F. nyanzae*	I 480	Zimbawbe	MT893586	-T . . . . . . . .	. . . . . . . . . .	. . . . . . . T . .	. . . . . . . . . .	365	47.95
*F. nyanzae*	I 394	Zimbabwe	MW046872	-T . . . . . . . .	. . . . . . . . . .	. . . . . . . T . .	. . . . . . . . . .	365	47.95
*F. nyanzae*	I 393	Zimbabwe	MW046870	-T . . . . . . . .	. . . . . . . . . .	. . . . . . . T . .	. . . . . . . . . .	365	47.95
*F. nyanzae*	I 2	Zimbabwe	MT909820	-T . . . . . . . .	. . . . . . . . . .	. . . . . . . T . .	. . . . . . . . . .	365	47.95
*F. nyanzae*	I 1	Zimbabwe	MT909821	-T . . . . . . . .	. . . . . . . . . .	. . . . . . . T . .	. . . . . . . . . .	365	47.95
*Fs. jacksoni*	Fsj-ITS1-A	Sri Lanka	ON661093	A ... A . . . . .	. . CG. . ACTC	TT-AG . A . . .	. . . . . TG. . T	364	48.90
*Fs. magna*	n.d.	Oregon, USA	KU232369	A . GT. GTT- -	- T . . CGTCTC	T. - GGG. . . C	C G . CCT. CT.	364	50.00
*Fs. magna*	n.d.	MS, USA	EF051080	A . GT. GTT- -	- T . . CGTCTC	T. - GGG. . CC	C G . CCT. CT.	364	50.00

Differences observed in a 433-bp-long alignment including 398 conserved and 35 variable positions plus 5 gapped positions, of which 34 were parsimony informative positions and 1 was a singleton site. Positions, numbers (to be read in vertical) refer to variable positions obtained in the alignment made with MEGA X; . = identical; - = insertion/deletion; n.d., not determined.

a, Andorra, France, Poland, Mexico, Venezuela, Ecuador, Peru, Bolivia, Uruguay, Argentina.

b, Niger, Nigeria, Senegal, southern Algeria, Cameroon.

K, Klaserie Private Nature Reserve; S, Sabi Sand Nature Reserve.

**Table 4:** Polymorphic sites in the sequence comparison of the nuclear rDNA ITS-2 between haplotypes of “genetically pure” *Fasciola hepatica* from livestock in Europe and the Americas, “genetically pure” *F. gigantica* from livestock in African countries, *F. nyanzae* from hippopotamuses in the nature reserves in South Africa, and from snails and one hippopotamus in Zimbabwe, *Fascioloides jacksoni* from elephants in Sri Lanka, and *Fs. magna* from cervids in USA.

**Fasciolid species**	**haplotype (H) or isolate (I)**	**Country**	**Acc. No**.	**ITS-2 variable positions**						**Length (bp)**	**GC (%)**
				11111	1111112222	2222222222	2222223333	3333333333	333		
				257822234	4558990001	3333455777	8888890001	2233333334	555		
				6779001232	6182340341	3579803234	0168913457	5801235897	169		
*F. hepatica*	Fh-ITS2-1	Spain + a	MG569980	T A T T G A T G T A	G G A T C T G A G T	C T T A A A T G T C	C A G C A T G A A G	C T T T C A A G C C	TGT	365	48.49
*F. hepatica*	Fh-ITS2-2	Spain + b	MG569981	. . . . . . . . . .	. . . . . . . . . .	. . . . . . . . . .	. . . T . . . . . .	. . . . . . . . . .	. . .	365	48.22
*F. hepatica*	Fh-ITS2-Htz*	Ecuador	MK212150	. . . . . . . . . .	. . . . . . . . . .	. . . . . . . . . .	. . . Y . . . . . .	. . . . . . . . . .	. . .	365	48.35
*F. gigantica*	Fg-ITS2-1	Burkina Faso + c	AJ853848	. . . . . . . . . .	. . . . . . . . . .	. C . . . . . . . T	T . . . . . . . . .	. . . - . . . A . .	. . .	364	48.08
*F. gigantica*	Fg-ITS2-2	southern Algeria	ON661090	. . . . . . . . . .	. . . . . . . . . .	. C . . C . . . T	T . . . . . . . . .	. . . - . . . A . .	. . .	364	48.35
*F. gigantica*	Fg-ITS2-Htz*	southern Algeria	ON661091	. . . . . . . . . .	. . . . . . . . . .	. C . . M . . . T	T . . . . . . . . .	. . . - . . . A . .	. . .	364	48.21
*F. nyanzae*	Fn-ITS2-1	South Africa-K	ON661092	. . . . . . . . . .	. . . . . . . . . .	. . . . . . . . . T	. . . . . . . . . .	. . . . . . . A . .	. . .	365	47.95
*F. nyanzae*	Fn-ITS2-1	South Africa-S	ON661092	. . . . . . . . . .	. . . . . . . . . .	. . . . . . . . . T	. . . . . . . . . .	. . . . . . . A . .	. . .	365	47.95
*F. nyanzae*	I S8bLNA1	Zimbabwe	MT893595	. . . . . . . . . .	. . . . . . . . . .	. . . . . . . . . T	. . . . . . . . . .	. . . . . . . A . .	. . .	365	47.95
*F. nyanzae*	I 483	Zimbabwe	MT893588	. . . . . . . . . .	. . . . . . . . . .	. . . . . . . . . T	. . . . . . . . . .	. . . . . . . A . .	. . .	365	47.95
*F. nyanzae*	I 481	Zimbabwe	MT893587	. . . . . . . . . .	. . . . . . . . . .	. . . . . . . . . T	. . . . . . . . . .	. . . . . . . A . .	. . .	365	47.95
*F. nyanzae*	I 480	Zimbabwe	MT893586	. . . . . . . . . .	. . . . . . . . . .	. . . . . . . . . T	. . . . . . . . . .	. . . . . . . A . .	. . .	365	47.95
*F. nyanzae*	I 394	Zimbabwe	MW046872	. . . . . . . . . .	. . . . . . . . . .	. . . . . . . . . T	. . . . . . . . . .	. . . . . . . A . .	. . .	365	47.95
*F. nyanzae*	I 393	Zimbabwe	MW046870	. . . . . . . . . .	. . . . . . . . . .	. . . . . . . . . T	. . . . . . . . . .	. . . . . . . A . .	. . .	365	47.95
*F. nyanzae*	I 1	Zimbabwe	MT909820	. . . . . . . . . .	. . . . . . . . . .	. . . . . . . . . T	. . . . . . . . . .	. . . . . . . A . .	. . .	365	47.95
*F. nyanzae*	I 2	Zimbawbe	MT909821	. . . . . . . . . .	. . . . . . . . . .	. . . . . . . . . T	. . . . . . . . . .	. . . . . . . A . .	. . .	365	47.95
*Fs. jacksoni*	Fsj-ITS2-1	Sri Lanka	ON661093	. T. - . GCA. G	TAGA . CA . TC	T . . GG . C . . T	TGA . GC . GG .	T . A . A . GAAT	GA.	364	48.90
*Fs. magna*	n.d.	Oregon, USA	KU232369	CTG- A. . . CG	. . GAACAGTG	T . C . . GCACT	. GA . GCAGGA	. CG . TGTAAT	. AA	364	50.00
*Fs. magna*	n.d.	MS, USA	EF051080	CTG- A. . . CG	. . GAACAGTG	T . C . . GCACT	. GA . GCAGGA	. CG . TGTAAT	. AA	364	50.00

Differences observed in a 365-bp-long alignment including 314 conserved and 51 variable positions plus 3 gapped positions, of which 50 were parsimony informative positions and 1 was a singleton site. Positions, numbers (to be read in vertical) refer to variable positions obtained in the alignment made with MEGA X; . = identical; - = insertion/deletion; n.d., not determined.

*Htz = pure species with heterozygotic position not differentiating between *F. hepatica* and *F. gigantica* (represented with corresponding symbol of IUPAC code for incomplete nucleic acid specification).

a, France, Poland, Mexico, Venezuela, Peru, Bolivia, Uruguay, Argentina.

b, Andorra, Mexico, Ecuador, Bolivia, Uruguay.

c, Niger, Nigeria, Senegal, Cameroon.

K, Klaserie Private Nature Reserve; S, Sabi Sand Nature Reserve.

**Table 5:** Estimates of evolutionary divergences over sequence pairs between species groups in the complete sequences of the nuclear rDNA and mtDNA markers.

**(A) rDNA ITS-1**				
*F. nyanzae*				
*F. gigantica*	3.0			
*F. hepatica*	2.0	4.4		
*Fs. jacksoni*	17.0	17.4	15.0	
*Fs. magna*	26.5	25.9	24.5	25.5
**(B) rDNA ITS-2**				
*F. nyanzae*				
*F. gigantica*	2.3			
*F. hepatica*	2.3	4.4		
*Fs. jacksoni*	32.0	30.3	34.3	
*Fs. magna*	37.0	37.3	39.3	29.0
**(C) Complete intergenic region**				
*F. nyanzae*				
*F. gigantica*	6.4			
*F. hepatica*	5.3	8.9		
*Fs. Jacksoni*	50.0	47.8	49.3	
*Fs. magna*	63.5	64.3	64.8	55.5
**(D) mtDNA *cox*1 gene**				
*F. nyanzae*				
*F. gigantica*	119.9			
*F. hepatica*	140.9	130.6		
*Fs. Jacksoni*	215.6	199.8	212.0	
*Fs. magna*	198.8	192.9	205.4	187.7
**(E) mtDNA *nad*1 gene**				
*F. nyanzae*				
*F. gigantica*	80.8			
*F. hepatica*	77.2	75.6		
*Fs. jacksoni*	140.9	132.0	133.6	
*Fs. magna*	136.4	133.4	136.6	106.0

The number of base differences (ts + tv) per sequence from averaging over all sequence pairs between groups are shown. All ambiguous positions were removed for each sequence pair. Evolutionary analyses conducted in MEGA X.

### Mitochondrial DNA gene sequences

The complete mtDNA *cox*1 gene provided seven different sequences with the same length of 1,533 bp and an average of AT content of 60.59%. Their alignment showed seven variable positions, including four parsimony-informative and three singleton, five of which close to the 3' end. The COX1 protein was 511 aa long, with start/stop codons of ATG/TAG, and provided three haplotypes. The differences between these three protein haplotypes were restricted to only one or two amino acid changes in the protein alignment (Table 6).

**Table 6:** Nucleotide and amino acid differences found in the complete mtDNA *cox*1 sequence of *Fasciola nyanzae* from South Africa and other *F. nyanzae* isolate fragments from Zimbabwe.

***F. nyanzae* haplotype (H) or isolate fragment (I)**	Locality^*^	**Country**	Host^**^	**GenBank Acc. No**.	***cox*** **1 nucleotide sequence**						**COXI amino acid sequence**	
						**Variable positions**				**Long (bp)**	**AT (%)**	**Variable positions**	**Length (aa)**
							11	1111111111			113443	
					1222233344	4445666666	8999999900	0000013445			6373499	
					9001300400	4492014569	0256669900	0111475992			8547089	
					5139116538	1450785199	7783690325	9148130364				
H cox1-a	K	South Africa	H.amph	ON661094	CGTCTTGATA	CTCCCTTGAT	CGCCGACCGG	AAGATGCATA	1,533	60.60	MWHMIHI	510
H cox1-b	K	South Africa	H.amph	ON661095	. . . . . . . . . .	. . . . . . . . . .	. . . . . . . . . .	. . . . . . . C . .	1,533	60.53	. . . . . P .	510
H cox1-c	S	South Africa	H.amph	ON661096	. . . . . . . . . .	. . . . . . . . . .	. . . . . . . . . .	. . . . . AT . AG	1,533	60.67	. . . . . . N	510
H cox1-d	S	South Africa	H.amph	ON661097	. . . . C . . . . .	. . . . . . . . . .	. . . . . . . . . .	. . . . . . . . AG	1,533	60.47	. . . . . . N	510
H cox1-e	S	South Africa	H.amph	ON661098	. . . . . . . . . .	. . . . . . . . . .	. . . . . . . . . .	. . . . . . . . AG	1,533	60.53	. . . . . . N	510
H cox1-f	S	South Africa	H.amph	ON661099	. . . . . . . . . .	. . . . . . . . . .	. . . . . . . . . .	. . . . . A . . AG	1,533	60.60	. . . . . . N	510
H cox1-g	K	South Africa	H.amph	ON661100	. . . . . . . . . .	. . . T . . . . . .	. . . . . . . . . .	. . . . . AT . AG	1,533	60.73	. . Y . . . N	510
I S8b LNA 1	Kariba	Zimbabwe	R.nat	MT909550	TA . . . . . . C .	. . . . TA- - - -	- - - - - - - - - -	- - - - - - - - - -	428	59.35	. R . - - - -	142
I KAR S3 RAD 10	Kariba	Zimbabwe	R.pli	MT909549	TAA . . . . C . .	. . . . T . CCG-	- - - - - - - - - -	- - - - - - - - - -	482	58.92	K . . - - - -	160
I 483	Kariba	Zimbabwe	R.nat	MT909548	TA . . . . . . . .	TC . . T- - - - -	- - - - - - - - - -	- - - - - - - - - -	425	59.29	. . . - - - -	141
I 480	Kariba	Zimbabwe	R.nat	MT909546	TA . . . . AG . G	T . T . . - - - - -	- - - - - - - - - -	- - - - - - - - - -	424	59.43	. . . - - - -	141
I 32	Kariba	Zimbabwe	P.col	MT909545	TA . T . . . . . .	. . . . T - - - - -	- - - - - - - - - -	- - - - - - - - - -	425	59.53	. . . - - - -	141
I 2	Kariba	Zimbabwe	H.amph	MT909543	- - - T . . . . . .	. . . . T . . TG	TA TGTTTTAA	GTTGG- - - - -	862	60.90	? . . VV -	287
I 1	Kariba	Zimbabwe	H.amph	MT909542	- - . . . C . . . .	. . . . . . . . GC	. . . T . . . . . .	. . . - - - - - - -	815	59.39	? . . . - - -	271

Positions, numbers (to be read in vertical) refer to variable positions obtained in the 1,533-bp-long alignment made with MEGA X; . = identical; shaded positions with a gap = not sequenced.

* K, = Klaserie Private Nature Reserve; S, = Sabi Sand Nature Reserve.

** H.amph, *Hippopotamus amphibius*; R.nat, *Radix natalensis*; R.pli, *Radix plicatula*; P.col, *Pseudosuccinea columella*.

When including the five 424–482-bp-long fragments of *F. nyanzae cox*1 obtained from lymnaeid snails and the two 815–862-bp-long fragments of the same gene obtained in only one hippopotamus, all from an artificial lake in Zimbabwe, a high number of 40 nucleotide differences appear in a 1,533-bp-long alignment (Table 6). None of the variable positions in the Zimbabwe sequences coincide with the only two observed in the corresponding fragment, and the sequence extreme where the five variable positions were detected in South African reserves was unfortunately not included in the Zimbabwe fragments.

Another alignment was performed including sequences from GenBank where appropriate comparison purposes, including the seven complete *cox*1 haplotypes of *F. nyanzae* from the South African reserves plus the complete sequences of *cox*1 of (i) 10 haplotypes of “genetically pure” *F. hepatica* from Europe and Latin America where *F. gigantica* is absent, (ii) 14 haplotypes of “genetically pure” *F. gigantica* from six African countries where *F. hepatica* is absent, (iii) eight isolates of *F. jacksoni* from Sri Lanka, and (iv) three isolates of *F. magna* from USA. The resulting alignment included a total of 42 sequences and was 1,546 pb long, of which 1,157 were conserved and 388 variable (359 p-info +29 singleton) (table not shown). This number of variable positions defines a genetic divergence of 25.1% for this *cox*1 gene in these fasciolids.

To assess the evolutionary speed of the *cox*1 gene inside the genus *Fasciola*, a genetic divergence of 14.36% was deduced from the 1,546-bp-long alignment with 222 variable positions including only the aforementioned 31 sequences of the three species of *Fasciola*. Additionally, a slightly smaller genetic divergence of 12.95% was deduced for *Fascioloides* from the 1,545-bp-long alignment with 200 variable positions including only the aforementioned 11 sequences of the two species of this genus. The mean number of base differences in *cox*1 between the species of *Fasciola* and *Fascioloides* are noted in Table 5D.

For its part, the mtDNA *nad*1 gene provided seven different sequence haplotypes with the same length of 903 bp and an average AT content of 61.50%. Their alignment showed 12 variable positions (9 p-info and three singleton). The NAD1 protein showed only one 301-aa-long haplotype with start/stop codons of GTG/TAG in all specimens analyzed, and provided three different haplotypes (Table 7).

**Table 7:** Nucleotide and amino acid differences found in the complete mtDNA *nad*1 sequence of *Fasciola nyanzae* from South Africa.

***F. nyanzae* haplotype (H)**	Locality^*^	Host^**^	**GenBank Acc. No**.	***nad*** **1 nucleotide sequence**				**NAD1 amino acid sequence**	
				**Variable positions**		**Length (bp)**	**AT (%)**	**Variable positions**	**Length (aa)**
				1111111245	88			1	
				5555666280	16			555557	
				1489013839	94			123450	
H nad1-a	S	H.amph	ON661077	GAATGGGTTC	CC	903	61.35	G S Y G E A	300
H nad1-b	S	H.amph	ON661078	. . . . . . . C . T	. .	903	61.35	. . . . . V	300
H nad1-c	S	H.amph	ON661079	. . . . . . . . CT	T .	903	61.46	. . . . . V	300
H nad1-d	K and S	H.amph	ON661080	. . . . . . . . . T	. .	903	61.46	. . . . . V	300
H nad1-e	K	H.amph	ON661081	TGTGTTC . . T	T .	903	61.68	WGLLQV	300
H nad1-f	K	H.amph	ON661082	TGTGTTCC . T	. .	903	61.46	WGLLQV	300
H nad1-g	K	H.amph	ON661083	TGTGTTC . . T	. .	903	61.57	WGLLQV	300

Positions = numbers (to be read in vertical) refer to variable positions obtained in the 903-bp-long alignment made with MEGA X; . = identical.

* K, Klaserie Private Nature Reserve; S, Sabi Sand Nature Reserve.

** H.amph, *Hippopotamus amphibius*.

Comparative *nad*1 alignment analyses were performed including our seven haplotypes of *F. nyanzae* with other complete sequences of *nad*1 gene, including (i) seven haplotypes of “genetically pure” *F. hepatica* from Europe and Latin America, (ii) 21 haplotypes of “genetically pure” *F. gigantica* from six African countries where *F. hepatica* is absent, (iii) eight isolates of *Fs. jacksoni* from Sri Lanka, and (iv) three isolates of *Fs. magna* from the USA. The resulting 903-pb-long alignment including these 46 sequences showed a length of 903 pb, of which 675 were conserved and 228 variable (224 p-info + 4 singleton) (table not shown).

Finally, to assess the evolutionary speed of the *nad*1 gene inside the genus *Fasciola*, a genetic divergence of 14.06% was deduced from the 903-bp-long alignment with 127 variable positions including only the aforementioned 35 sequences of the three species of *Fasciola*. Additionally, a similar genetic divergence of 14.16% was deduced for *Fascioloides* from the 903-bp-long alignment with 112 variable positions including only the aforementioned 11 sequences of the two species of this genus. The mean number of base differences in *nad*1 between the species of *Fasciola* and *Fascioloides* are noted in Table 5E.

### Phylogenetic analyses

**Figure 3:**
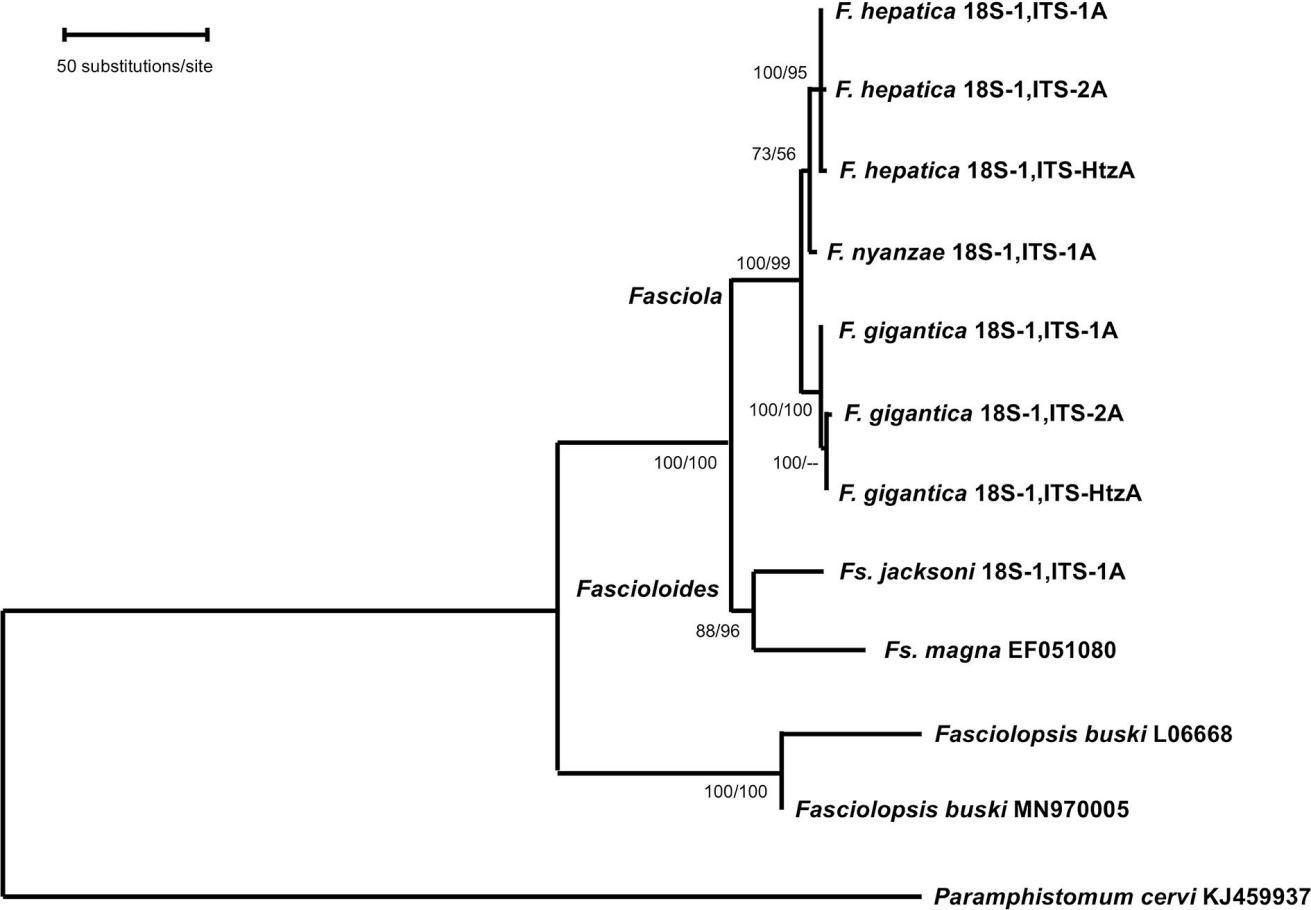
Phylogenetic tree of species of *Fasciola, Fascioloides*, and *Fasciolopsis* based on maximum-likelihood (ML) estimates and reconstructed on the concatenated sequences of the nuclear rDNA 18S gene, ITS-1, 5.8S gene and ITS-2 (lnL = −7,832.871). The tree is drawn to scale, with branch lengths measured in the number of substitutions per site. The tree was rooted using the sequence of *Paramphistomum cervi* (KJ459937) as outgroup. Supports for nodes a/b using MEGA X: a, bootstrap (1,000 replicates) with ML parameters (HKY + G); b, bootstrap (1,000 replicates) with NJ and number of differences method for distances.

Phylogenetic analysis carried out with the combination of the 18S rRNA gene and the complete intergenic region of the ribosomal operon, including ITS-1, 5.8S and ITS-2 in a single data-set, generated a robust tree, indicating phylogenetic accordance between the ribosomal markers. The ML model best fitting this data-set was Hasegawa-Kishino-Yano model with discrete Gamma distribution (HKY + G), using a ts/tv ratio of 2,183 (LnL = −7832.871), and a +G, parameter = 0.243. In the ML tree obtained (Figure 3), the three *Fasciola* species cluster together with high support values (100/99 in ML/NJ), appearing *F. nyanzae* as a sister species to the clade of *F. hepatica*. The two *Fascioloides* species (*Fs. jacksoni and Fs. magna*), also cluster together, and the big clade, including both genera, *Fasciola and Fascioloides*, show maximum support (100/100 in ML/NJ). Although clustering in the same clade, a clear phylogenetic distance is observed between the two *Fasciolopsis buski* sequences appearing basal to the two groupings of *Fasciola* and *Fascioloides*. The topology obtained with the NJ algorithm using the number of differences method (figure not shown) was identical to that shown by the ML tree.

**Figure 4:**
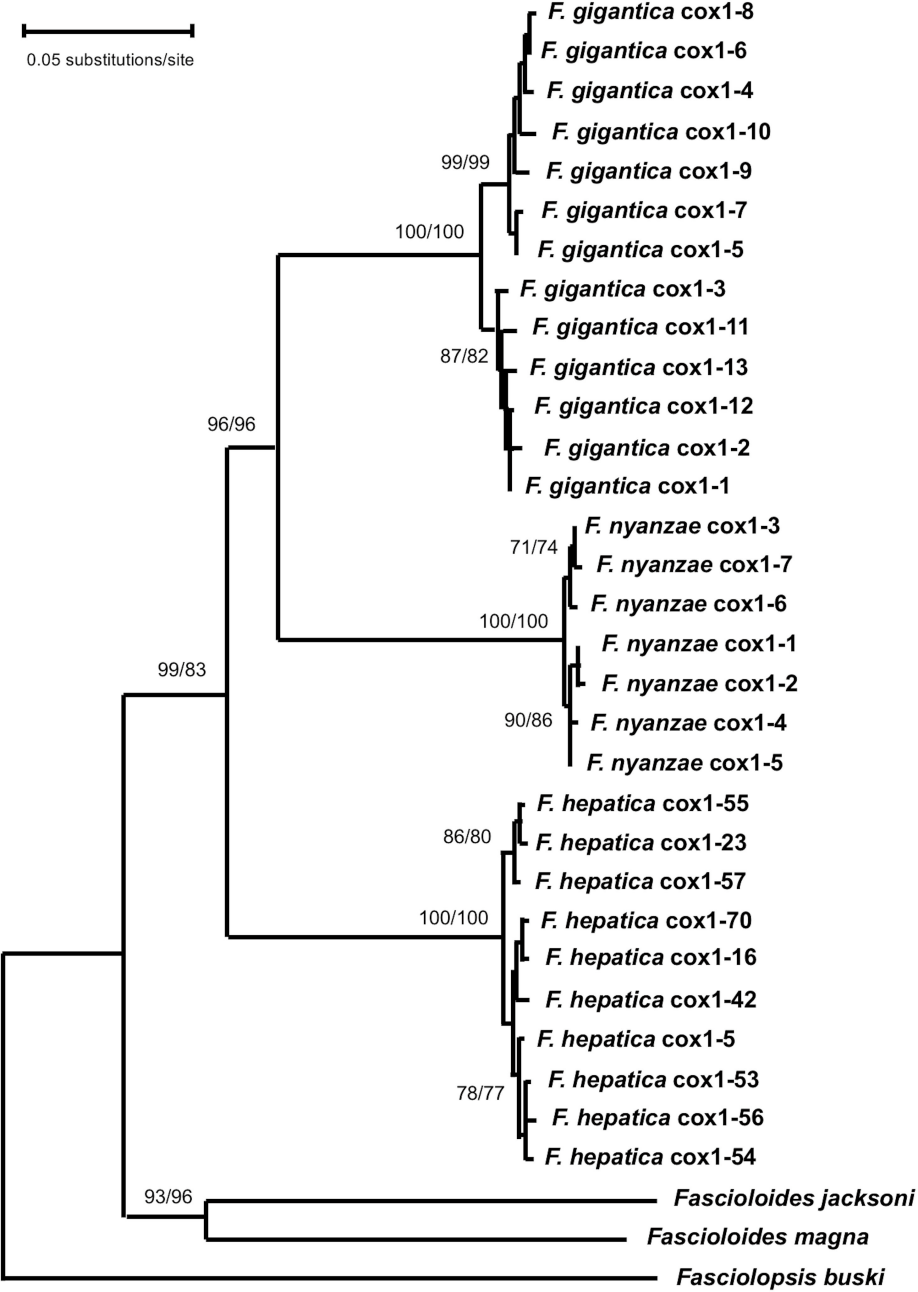
Phylogenetic tree of species of *Fasciola* and *Fascioloides* based on maximum-likelihood (ML) estimates and reconstructed on mtDNA *cox*1 sequences (LnL = −5,733.3311). The tree is drawn to scale, with branch lengths measured in the number of substitutions per site. The tree was rooted using the sequence of *Fasciolopsis buski* (MF287794) as outgroup. Supports for nodes a/b using MEGA X: a, bootstrap (1,000 replicates) with ML parameters (T92 + G); b, bootstrap (1,000 replicates) with NJ and Tamura 3-p method.

For the mtDNA *cox*1 data matrix, the best fitting model was Tamura 3-parameter (T92 + G) with discrete Gamma distribution (T92 + G). The resulting ML tree (log likelihood = −5,733.3311) was inferred with +*G*, parameter = 0.4351 and the rate variation model allowed for some sites to be evolutionarily invariable (Figure 4). In the ML tree, the seven haplotypes of *F. nyanzae* cluster together in a monophyletic clade, closely related and well-supported (96/96 in ML/NJ) with *F. gigantica* clade. Thus, all haplotypes from different African countries grouped together in a monophyletic clade. The haplotypes of *F. hepatica* from Europe and Latin American countries appear in a basal position regarding *F. nyanzae* and *F. hepatica*, all included in a single clade of the genus *Fasciola* with a high support (99/83 in ML/NJ). The two *Fascioloides* species (*Fs. jacksoni* and *Fs. magna*) clustered together in an independent clade also with a very high support (93/96 in ML/NJ). With the NJ algorithm, and using the Tamura 3-parameter method, identical topology of the ML tree was obtained (tree not shown).

**Figure 5:**
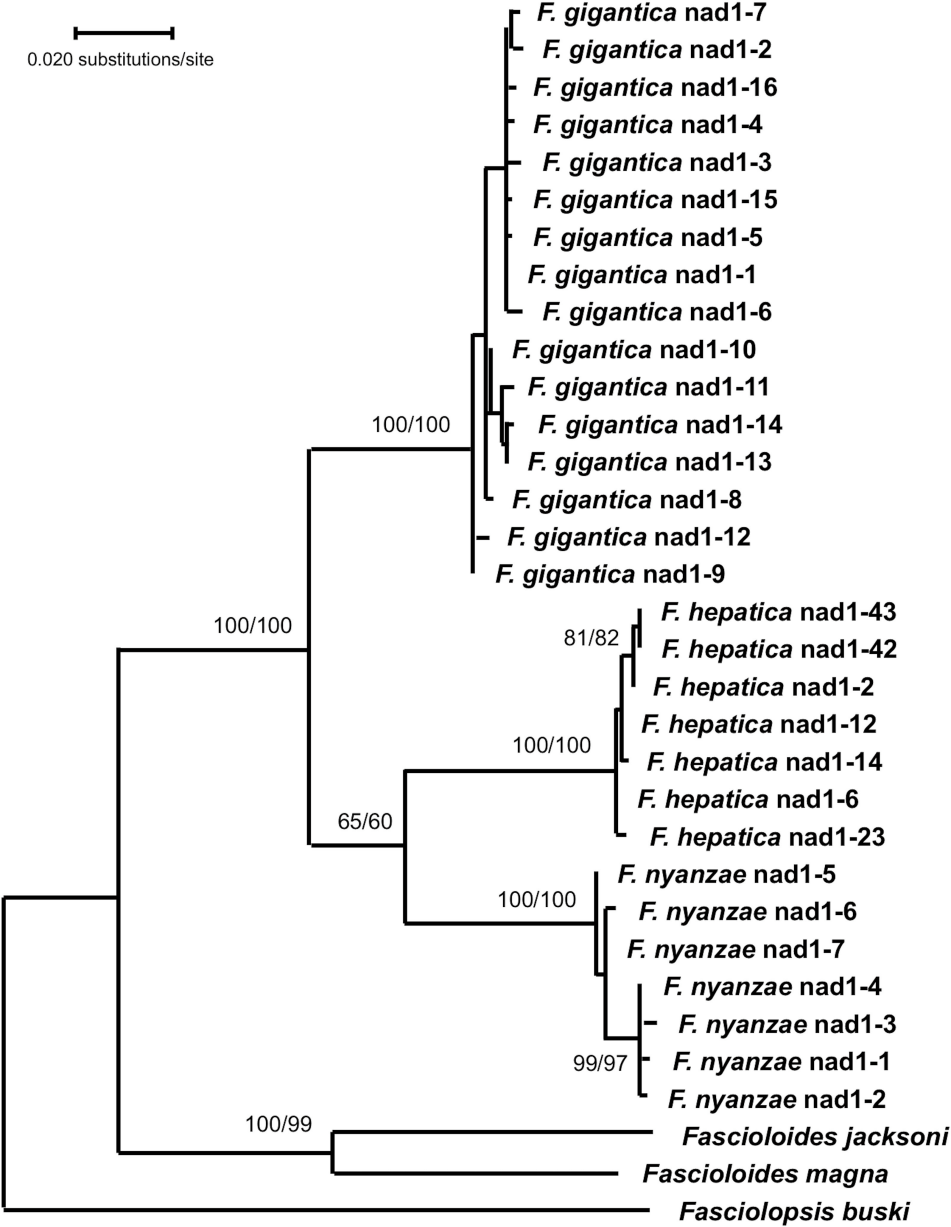
Phylogenetic tree of species of *Fasciola* and *Fascioloides* based on maximum-likelihood (ML) estimates and reconstructed on mtDNA *nad*1 sequences (LnL = −3559.05). The tree is drawn to scale, with branch lengths measured in the number of substitutions per site. The tree was rooted using the sequence of *Fasciolopsis buski* (MF287793) as outgroup. Supports for nodes a/b using MEGA X: a, bootstrap (1,000 replicates) with ML parameters (T92 + G); b, bootstrap (1,000 replicates) with NJ and Tamura 3-p method.

In the phylogenetic analysis carried out with the mtDNA *nad*1 data matrix, the ML model best fitting this data-set was T92 + G. The tree with the highest log likelihood (−3,559.05) was constructed with the rate variation among sites modeled with a gamma distribution (+*G*, parameter = 0.3091) (Figure 5). The ML topology obtained shows a monophyletic clade for the three *Fasciola* species with the highest supports (100/100 in ML/NJ). The two species of *Fascioloides* clustered together in another clade also with a very high support (100/99 in ML/NJ), thus manifesting its clear independence from that of the *Fasciola* clade. With the NJ algorithm, and using the Tamura 3-p method, identical topology of the ML tree was obtained (tree not shown).

All topologies obtained with ribosomal and mitochondrial markers were solid, well-supported and congruent, corroborating the phylogenetic relationship of *F. nyanzae* within the genus *Fasciola*. Moreover, the three phylogenies agree in the inclusion of the species *Fs. jacksoni* and *Fs. magna* in the genus *Fascioloides* and its distant relationship with the genus *Fasciola*.

## Discussion

### *Fasciola nyanzae* phenotypic and genotypic comparison

*Fasciola nyanzae* is a parasite specific of the bile ducts of the hippopotamus. No adult flukes were produced in experimental infections of calves, goats and rabbits (29), and it was never reported in cattle around areas where it is frequent in Uganda (47). Parasitological studies on hippopotamuses have reported the parasite several times in Uganda (48, 49), Kenya (50), South Africa (51), the southern part of Chad (52), Gabon (53), and in an artificial lake in Zimbabwe (30, 54). These findings indicate a very wide distribution throughout Africa, which overlaps with that of its specific freshwater lymnaeid snail vector species *R. natalensis* (29) (Figure 2).

The species markedly differs morphologically from *F. hepatica* and *F. gigantica* by its length, the great breadth of the shoulders, the large cone, and the long tapering body, almost entirely occupied by vitelline follicles, with the distance between the posterior testis and the posterior extremity of the body being longer than the remaining anterior part of the body (Figure 1). The maximum length of the adult stage infecting the hippopotamus has been noted to reach 69 mm (55), and up to 91 mm (29), but reached even 136.6 mm in the longest specimen from the South African reserves. The adult maximum length is only around 55 mm in *F. gigantica* and only 31 mm in *F. hepatica* (25, 56). However, egg size overlap: *F. nyanzae* eggs measure 150–190/70–110 μm in the uterus (50), 132–150/79–97 μm in bile, and 131–160/75–88 μm in specimens mounted in Canada balsam (29); *F. gigantica* eggs are of 130.3–182.8/74.0–123.6 μm in animals and 150.9–182.2/85.1–106.2 μm in humans; and F. hepatica eggs range 73.8–156.8/58.1–98.1 μm in animals and 100.6–162.2/65.9–104.6 μm in humans (5, 57).

In the 18S rDNA gene, the few mutations between the three species of *Fasciola* and the two species of *Fascioloides* agree with the conserved characteristics of this gene (26). The sequence of ITS-1 of *F. nyanzae* from hippopotamus in the South African reserves is identical to that obtained in snails and in one hippopotamus in an artificial lake of Zimbabwe (30).

The numerous mutations in the short *cox*1 fragments obtained from *F. nyanzae* in Zimbabwe (30, 54) represent a surprising intraspecific variability for specimens from only one place and are difficult to interpret in materials from an artificial lake. Therefore, these numerous mutations should be individually verified in the electropherograms and the geographical origin of the snail and hippopotamus hosts introduced in the artificial lake should be analyzed regarding the sequences in question.

In the phylogenetic tree obtained with the concatenated rDNA sequences, the Asian species *Fasciolopsis buski* proves to be phylogenetically very distant from the species of *Fasciola* and *Fascioloides*. This result agrees with the completely different characteristics of *Fps. buski* when compared to the other fasciolids, including the morpho-anatomy of its adult stage with non-ramified intestinal caeca (vs. ramified in the other fasciolids), the intestinal microhabitat of infection (vs. a liver microhabitat), the omnivore definitive host (vs. herbivores), and planorbids as snail vectors (vs. lymnaeids) (45). This indicates that the genus *Fasciolopsis* has no close evolutionary relationship with the genera *Fasciola* and *Fascioloides* and may consequently be ruled out when analyzing the direct derivation of the paleobiogeographical origins of *F. hepatica* and *F. gigantica*. The species *Fps. buski* has been therefore used as outgroup for the two phylogenetic reconstructions by means of the *cox*1 and *nad*1 genes to obtain better trees than with the more distant *P. cervi*.

Both sequence analyses and phylogenetic trees evidence (i) the close relationships of *F. nyanzae* with *F. gigantica* and *F. hepatica* and consequently its belonging to the genus *Fasciola*, and (ii) the distant relationships of *Fasciola* with the two species *Fs. jacksoni* and *Fs. magna* included in the genus *Fascioloides*.

### Divergence estimates

Trematodes do not fossilize, but their evolution may be estimated considering the fossil record of their specific, co-evolving definitive hosts and snail vectors (58). Hippopotamid and ruminant hosts of the three *Fasciola* species have evolved between the Early Miocene and the Pleistocene (59–61), whereas lymnaeid snails are pronouncedly older, follow a slower evolution, and their oldest fossil record dates back to the Jurassic time period (62). When dating estimates of relative divergences in such past periods, the conserved nuclear rDNA 18S gene becomes appropriate because (i) it allowed for a reliable alignment of the sequences across the taxa analyzed, (ii) it follows a low mutation rate in both fasciolids and lymnaeids (63), (iii) it is a non-coding gene, (iv) general traits and length of the phases of the life cycle of *Fasciola* (64) and *Fascioloides* (22) are very similar and timely overlap, indicating a similar number of generations along time and therefore a similar evolutionary speed rate, and (v) snail vectors of *Fasciola* and *Fascioloides* belong to the same or similar species. The last two factors exclude the need to apply different evolutionary rates for the lineages of *Fasciola* and *Fascioloides*. As it has been highlighted, when a constancy rate is not rejected, the use of a strict clock is preferred because it has the fewest parameters and generally leads to the most precise divergence estimates (65). Moreover, given that only three species of *Fasciola* and two species of *Fascioloides* are used for the analysis, this molecular clock meets the premise of a simple model which captures the essential biological features of the data. Recent studies have shown how increasing the data set size lead to increasing estimate errors, even in data sets of moderate size (65). Additionally, the relative divergence estimates obtained for fasciolids proved to fit very well with the fossil records of the oldest representatives of the definitive host groups and their subsequent intercontinental migrations, as well as with available knowledge on paleobiogeographical changes of the landscape, mainly concerning interconnecting bridges between continents.

When applying the conventional molecular clock rate of 1.8 × 10^−10^ substitutions per site per year (1.8% per 100 my) for the evolution of the 18S gene obtained in many studies of different organism groups which count on well-dated fossil remains (66), the time elapsed between the appearance of *F. gigantica* and *F. hepatica* by derivation from *F. nyanzae* is estimated to be around 8.41 my. If the oldest hippopotamus fossil record in Asia, of 6.1–5.9 mya (67–69), is considered, the appearance of *F. gigantica* should be rolled back to at least around 14.5 mya. This fits the upper limit marked by the appearance of the first primitive hippopotamids, dated back to 21.0–15.9 mya in the Early Miocene in Africa (56, 60). When analyzing the different phases of the split of the bovids along the Miocene (61), these data suggest that the capture phenomenon from early hippopotamids to ancient bovids grazing in flooded plains and lowlands close to freshwater collections inhabited by hippopotamuses most probably occurred during the third split phase corresponding to the radiation of mainly Reduncinae and when several ancestors of Bovinae and Alcelaphinae were also present in Africa, at about 13.5 mya. These evolutionary estimates indicate a time frame more recent than the 19 mya divergence obtained according to sequence analysis of cathepsin L-like cysteine proteases (70). However, subsequent assessments have demonstrated that metabolic rates are not appropriate for the calibration of the molecular clock (71).

The 18S molecular clock estimates the divergence between *Fs. magna* of cervids and caribou in North America and *Fs. jacksoni* of the Asian elephant at about 19.77 mya. This fits well with the faunal exchange between the Palearctic and Nearctic regions during which ancestral odocoileine cervids entered America from Siberia *via* the Bering Strait in the late Miocene/early Pliocene (72).

The divergence between *F. nyanzae* and *Fs. jacksoni* is of 22.44 mya and that between *F. nyanzae* and *Fs. magna* of 25.48 mya, whereas that between *F. gigantica* and *Fs. jacksoni* dates back to 22.44 mya and that between *F. gigantica* and *Fs. magna* to 28.31 mya. This indicates that the divergence between ancestral *Fasciola* and *Fascioloides* rolls back to the Late Oligocene-Earliest Miocene, pronouncedly before the appearance of *F. gigantica* and *F. hepatica*. Therefore, a direct derivation of the current species of *Fasciola* from *Fascioloides* does not appear to be supported.

Additional molecular clock divergence estimations based on the rDNA 18S gene were analyzed on lymnaeids (52) to verify that the snail vectors of the three *Fasciola* species were there in the past to assure the respective fasciolid life cycles. The molecular clock furnishes a divergence of 61.1 my between *G. truncatula* and *R. auricularia*, of 39.0 my between *R. natalensis* and *R. auricularia*, and of 0.33 my between *R. natalensis* of Burkina Faso and *R. natalensis* of Egypt. These dating estimates agree with the very old history and slow evolutionary rates of Lymnaeidae, whose oldest fossil record dates back to around 208–146 mya (62). The divergence between *Galba* and *Radix* appears to be very old, although it occurred pronouncedly later then the Gondwana division leading to the separation of the landmasses of South America and Africa by the Atlantic Ocean according to a very long process lasting between around 130 and 85 mya (73), which explains the absence of *Radix* in the New World. The time of divergence between *R. natalensis* and *R. auricularia* indicates that the former, the only *Radix* species present in the whole sub-Saharan region, became isolated in Africa from all other Palaearctic *Radix* species even before the Oligocene. This is sufficient time to expand passively transported by watercourses and ancient animals and cover wide zones of this continent. Concerning the time needed for *R. auricularia* to expand from the sub-Saharan region up to Egypt, is should be considered that the Nile river flow is indiscernibly slow and almost inexistent and that consequently it is more logical to think that this snail species should have been passively transported by animals, hippopotamuses included, following the river basin northward.

### Past and present distribution of hippopotamuses

The African *Hipppotamus amphibious* is at present showing a very wide geographical distribution covering whole Africa southward from the Sahara desert (74), according to a markedly fragmented presence of populations linked to the peaceful type of water collections it inhabits. Hippopotamuses are amphibious herbivorous mammals which leave water to graze vegetation in the surroundings over night (75), a behavior which facilitates its infection by fasciolid flukes. The oldest fossils of hippopotamuses are dated back to the Early Miocene and indicate that these mammals reached their major diversity in sub-Saharan Africa afterwards during the Plio-Pleistocene (76). Molecular assessments suggest that their original area might have been in eastern Africa, around present-day Uganda, southwestern Kenya, eastern Congo, Tanzania, Zambia, and northern Zimbabwe (77) (Figure 2). Paleontological data indicate that hippopotamuses spread northwards to North Africa and Europe during the late Middle or Late Pleistocene (76).

**Figure 6:**
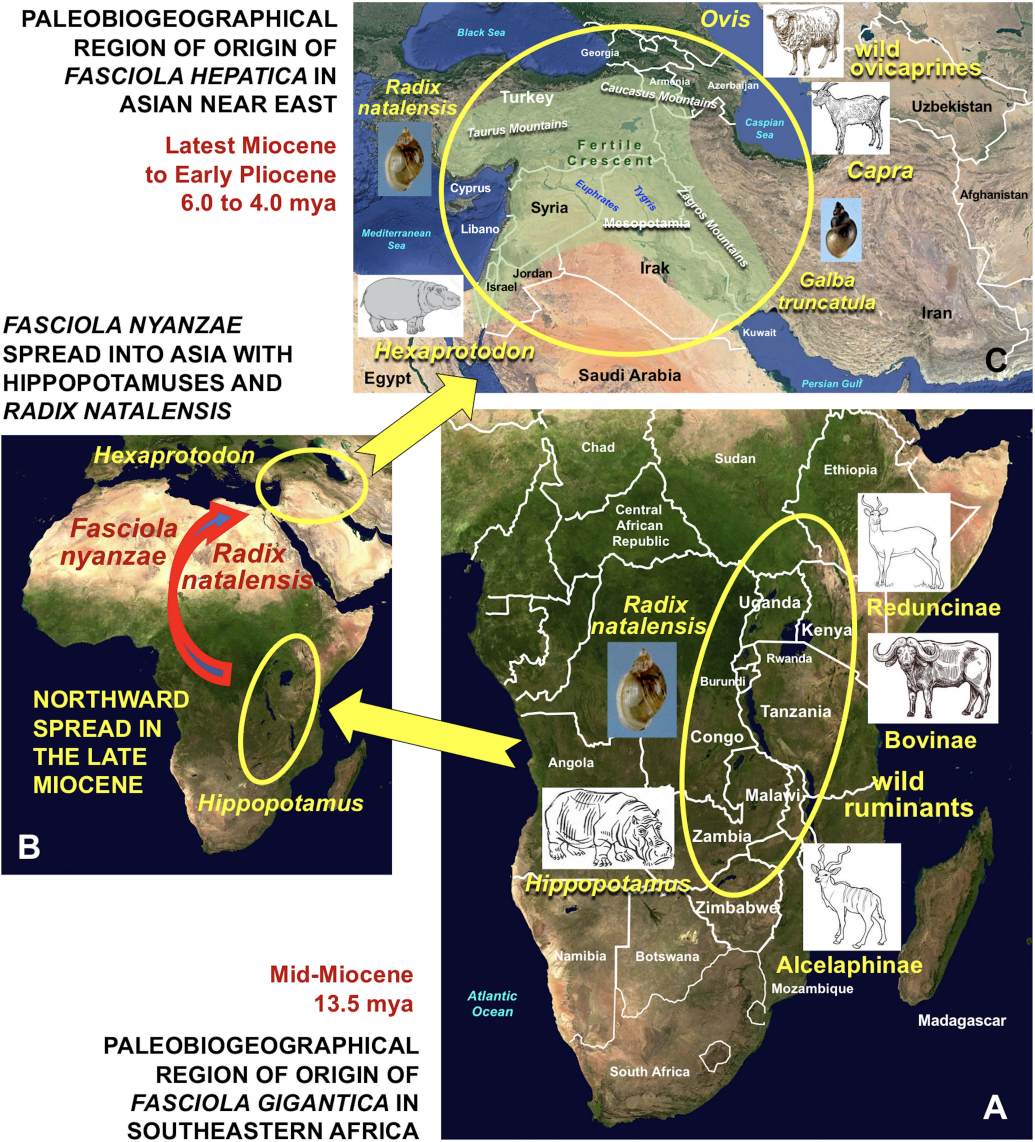
Paleobiogeographical regions of origin of *Fasciola gigantica* and *F. hepatica* by direct evolutionary derivation from *F. nyanzae* infecting past hippopotamuses. **(A)** Origin of *F. gigantica* in southeastern Africa by definitive host capture phenomenon from African past hippopotamuses to wild ruminants, mainly ancestors of the bovid subfamilies Reduncinae, Bovinae and Alcelaphinae by keeping the same snail vector *Radix natalensis* in the Mid-Miocene, around 13.5 mya; **(B)** northward spread of *F. nyanzae* with African hippopotamuses and *R. natalensis* and introduction from Africa into the Asian Near East with *Hexaprotodon* in the Late Miocene, around 6.1 and 5.9 mya; **(C)** origin of *F. hepatica* in Near East Asia by definitive host capture phenomenon from *Hexaprotodon* to wild species of ovicaprines of the genera *Ovis* and *Capra* and vector capture event from *R. natalensis* to *Galba truncatula* in the Latest Miocene to Early Pliocene, around 6.0–4.0 mya. Countries and geographic names are only those noted in the text. Geographic background from composed satellite maps of orthographic projections by NASA (public domain) *via* Wikimedia Commons. Schema S. Mas-Coma.

This northward spread led hippopotamuses to expand throughout southern Asia giving rise to the Asian fossil lineage included in the genus *Hexaprotodon* (Figure 6), which eastwards further reached the Indian subcontinent and up to South East Asia (76). The oldest fossils of Asian *Hexaprotodon* were found in the Siwalik hills of the present-day state of Himachal Pradesh in northeastern India, neighboring northern Pakistan and were dated back to the latest Miocene to Early Pliocene, between 6.1 and 5.9 mya (67–69). The passage from northeastern Africa into westernmost Asia probably occurred along the Levantine corridor. This is suggested by hippopotamid fossil remains found in present-day Georgia around 1.40 mya, in the Israel-Libano area around 1.40–0.70 mya, and in Syria around 0.30–0.25 mya (76). The present *H. amphibius* was still present in Palestina in the Neolithic and even until very recently in the northern Nile river basin (78).

In the African hippopotamuses, the orbits are elevated above the skull, and their nostrils and ears can be closed while diving, whereas eyes are non- or less-elevated in the Asian *Hexaprotodon* indicating a less amphibious, i.e., more terrestrial, life style (68).

### Past and present distribution of *Radix natalensis*

The snail *R. natalensis* is the only autochthonous lymnaeid in Africa southward of the Sahara desert. In this very wide region, other lymnaeids reported are all introduced species, whether recently (*Pseudosuccinae columella, R. rubiginosa, R. plicata, G. truncatula*) or evolutionarily recently (*G. mweruensis* in isolated refugia).

This freshwater snail shows a wide geographical distribution throughout the aforementioned sub-Saharan Africa, which covers that of the present populations of hippopotamus (79) (Figure 2). Three additional aspects of the geographical distribution of *R. natalensis* should be highlighted:

(A) The findings of fossilized shells in Late Pleistocene-Holocene sites throughout the Sahara desert (80) (Figure 2) speak about the past presence of watercourses along this desert (81), and indicate that this snail was accompanying hippopotamuses during their northward spread during the late Middle or Late Pleistocene (76).
(B) The distribution of *R. natalensis* includes a northward arm extending along the Nile river basin up to the Nile Delta at the Mediterranean shore (79) (Figure 2).
(C) Additional populations have been found in Jordan found in Azraq oasis and the Jordan Valley (82), and in Kishda, Palestina (83) (Figure 2), which indicate that this snail also reached western Asia, most probably accompanying the aforementioned past spread of hippopotamuses along the Levantine corridor (76).

*Radix natalensis* is a freshwater snail which inhabits steady water habitats and swampy areas with slow-flowing streams and canals usually with abundant aquatic vegetation in warm lowlands. The adaptability of *R. natalensis* to varying conditions of temperature (84, 85) and dryness (86, 87) underlie the persistence of this radicine lymnaeid over time once it colonizes and establishes in a freshwater habitat. In this way, this snail finds its ideal habitat in the same type of water habitats as the hippopotamus. This explains the sharing of the same sites by hippopotamuses and *R. natalensis*. This coexistence allows the life cycle of *F. nyanzae* to be established and maintained, as indeed *F. nyanzae* was experimentally demonstrated to be transmitted by *R. natalensis* (29), and the larval stages of this fasciolid have also been found in naturally infected specimens of this radicine lymnaeid (30, 54).

Worth mentioning is the fact that *R. natalensis* also acts as the specific transmitter of *F. gigantica* in Africa. To date, populations of *R. natalensis* from different countries have always demonstrated to be experimentally susceptible to the infection and able to transmit the many geographical strains of African *F. gigantica* assayed (88–96). Moreover, in the Levantine corridor in westernmost Asia, where it was referred to as *Lymnaea auricularia* in initial reports (97), the *R. natalensis* population of the Azraq oasis in Jordan (Figure 2) also showed to transmit the local *F. gigantica* (98, 99).

Finally, it should be highlighted that *R. natalensis* from Egypt has experimentally demonstrated to also transmit *F. hepatica* from Europe (100). This means that *R. natalensis* is able to transmit the three species of *Fasciola* and might be interpreted as this lymnaeid species being a kind of “mother vector” for these trematodes.

### *Fasciola gigantica* in the wild fauna in eastern Africa

Ungulates are hoofed mammals known to present their higher diversity in the savannah biome of sub-Saharan Africa. The analysis of population genetic data showed a concordance of the phylogeographic structuring of many ungulate species (77). The cradle of ungulate diversity appears in East Africa (101), where major biogeographic lineages of these herbivore mammals meet and overlap, including the hippopotamus.

Reports on *F. gigantica* infection in ungulates are numerous in several countries of eastern Africa (48, 102–105), including many different mammal species (Table 8). Five aspects should be highlighted:

(A) Although in most cases the coexistence with domestic ruminants indicates livestock to be the infection source for the sylvatic mammals due to the sharing of the same grazing areas, there have been reports in which it was specified that the infected wild animals had never been in contact with domestic ruminants, such as in cases of the Kob *Kobus kob*, the Hartebeest *Alcelaphus buselaphus*, and the buffalo *Syncerus caffer* in Uganda, or cases in which such a contact was noted to have been rare, as in cases of the buffalo in Uganda and Tanzania and the Eland *Taurotragus oryx* in Tanzania (48).
(B) Many of the wild ungulate species show major biogeographic patterns inferred from phylogeographic data which are found in eastern Africa coinciding with that of the hippopotamus (77).
(C) Several of the wild species found infected appear repeatedly in the different countries, such as the Hartebeest, Waterbuck, Kob, Topi, buffalo, Eland, Impala, and Wildebeest (Table 8), indicating a grazing behavior in appropriate areas for the infection by *F. gigantica*, i.e., where freshwater collections inhabited by *R. natalensis* are present.
(D) Very high prevalences were reported from the African buffalo (58%), kob *K. kob* (47%), and Hartebeest *A. buselaphus* (47%) in Uganda (103). as well as in the Kafue lechwe (52.5%) in Zambia and the Kudu (12.5%) in Zimbabwe (105).
(E) Infection intensities may reach high burdens in several of these wild animals, as it has been observed in the African buffalo (up to 66 flukes per host individual), kob *K. kob* (17 flukes) and Jackson's hartebeest *A. buselaphus* (21 flukes) in Uganda (103), and also in giraffe (41 flukes) from Kenya and Topi (30 flukes) in Tanzania (48).

**Table 8:** Species of the wild fauna of Africa in which infection by *Fasciola gigantica* has been reported.

**Chad**	**Rwanda**
*Alcelaphus buselaphus (Alcelaphinae) *-* Hartebeest *Kobus defassa (Reduncinae) *-* Waterbuck *Kobus kob (Reduncinae) – Kob **Sudan** *Damaliscus korrigum (Alcelaphinae) *-* Topi *Syncerus caffer (Bovinae) *-* buffalo **Central African Republic** *Syncerus caffer (Bovinae) *-* buffalo **Congo** *Alcelaphus buselaphus (Alcelaphinae) *-* Hartebeest *Kobus defassa (Reduncinae) *-* Waterbuck *Kobus kob (Reduncinae) *-* Kob **Uganda** *Alcelaphus buselaphus (Alcelaphinae) *-* Hartebeest *Kobus kob (Reduncinae) *-* Kob *Syncerus caffer (Bovinae) *-* buffalo *Taurotragus oryx (Bovinae) *-* Eland **Kenya** *Aepyceros melampus (Aepycerotinae) *-* Impala *Connochaetes taurinus (Alcelaphinae) *-* Wildebeest *Giraffa camelopardalis (Giraffidae) *-* Giraffe *Taurotragus oryx (Bovinae) *-* Eland	*Sylvicapra grimmia* (Cephalophinae) *-* Common or Gray duiker **Tanzania** *Connochaetes taurinus (Alcelaphinae) *-* Wildebeest *Damaliscus korrigum (Alcelaphinae) *-* Topi *Syncerus caffer (Bovinae) *-* buffalo *Taurotragus oryx (Bovinae) *-* Eland **Zambia** * [*Hippopotamus amphibius* (Hippopotamdae) - hippopotamus]** Kobus leche (Reduncinae) *-* Kafue lechwe *Kobus vardoni* (Reduncinae) *-* Puku **Zimbabwe** *Aepyceros melampus (Aepycerotinae) *-* Impala *Connochaetes taurinus (Alcelaphinae) *-* Wildebeest *Damaliscus lunatus* (Alcelaphinae) *-* Tsessebe or sassaby *Giraffa camelopardalis (Giraffidae) *-* Giraffe *Hippotragus niger (Hippotraginae) *-* Sable antelope *Redunca arundinum* (Reduncinae) *-* Reedbuck *Sylvicapra grimmia* (Cephalophinae) *-* Gray duiker *Taurotragus oryx (Bovinae) *-* Eland *Tragelaphus scriptus* (Bovinae) *-* Bushbuck *Tragelaphus strepsiceros (Bovinae) *-* Kudu **Swaziland** *Aepyceros melampus (Aepycerotinae) *-* Impala

Subfamilies are noted in the case of Bovidae.

*Ungulate species whose major biogeographic pattern inferred from phylogeographic data is found in eastern Africa coinciding with that of the hippopotamus.

**Finding in hippopotamus in Zambia initially reported as *Fasciola* sp. and later ascribed to *F. gigantica* by Round in 1968 (101) still remains a question mark.

It is evident that all necessary features for a host capture event of the large *F. nyanzae* from the big amphibious hippopotamus to smaller terrestrial ungulates were converging in eastern Africa in the past. This host capture phenomenon should have been the origin of speciation by adaptation to the new hosts giving rise to the smaller *F. gigantica* (Figure 6).

### *Fasciola hepatica* in the wild fauna scenario of the Near East

The westernmost Asian region of the Near East is unique in presenting the geographic and timely convergence of the following mammal hosts, lymnaeid snail vectors, fasciolid flukes, appropriate altitude-varying milieu, and climate characteristics:

- *F. gigantica* and *F. hepatica*;
- small-sized ruminants as sheep and goats;
- large sized ruminants as taurine cattle, zebu and buffalo;
- equines as donkeys, mules and horses, also including onager donkeys in the past;
- omnivores as wild swine;
- Old World camelids as Bactrian camel and dromedary;
- *R. natalensis, R. auricularia* and *G. truncatula*;
- past existence of hippopotamuses presumably harboring *F. nyanzae*;
- warm flat lowlands and cool mountainous highlands.

It was in this region where the so-called Fertile Crescent emerged and the domestication of wild herbivores began around 12,000–10,000 years ago (106–109), marking the dawn of the Neolithic period. The Fertile Crescent was a fertile zone for agricultural use covering from present-day Syria, Lebanon, Jordan, Israel and Palestine in the so-called Levant and part of Anatolia (present-day Turkey) including the Taurus mountains, in the West, along the Tigris and Euphrates river basins in the Mesopotamia flatlands and northward reaching the Caucasus mountains, and up to the Zagros mountains in present-day Irak and Iran, in the East.

Archeological excavations in a cave in the western-central Zagros allowed for the finding of remains of bovids, cervids, suids and equines dating back to around 75,000 years BP (110). This indicates that potential hosts for fasciolids were present in this region in the past. Comparative data on infectivity, life span, egg shedding and immunity indicate that sheep and goats are the ideal hosts of *F. hepatica* (1, 53). Ovicaprines are mid-sized ruminants which should therefore be considered among the original hosts of the smaller *F. hepatica*. A definitive host capture phenomenon from a *F. nyanzae* lineage in the less amphibious southwest Asian *Hexaprotodon* hippopotamuses toward a more terrestrial ovicaprine/*G. truncatula* binome may be envisaged to have occurred in Near East Asia around the latest Miocene - earliest Pliocene period.

Species of the two genera *Capra* and *Ovis* diversified around 6.8–5.1 mya (61). The wild ancestor of the domesticated goat *Capra hircus* was the bezoar *Capra aegagrus*, which was widely distributed from the Taurus mountains and eastern Anatolia in the West, down to the Iranian plateau in the East, through the Zagros mountainous chain along present Irak and Iran (111, 112). The wild ancestor of the domesticated sheep *Ovis aries* was the mouflon *Ovis gmelini* (= urial sheep *O. orientalis*) (113, 114), whose distribution covered the region from the southern Levant through southeastern Anatolia and northern Syria, to the high Zagros mountain pastures and lowland plains of Irak and Iran (108, 109). Indeed, the biome inhabited by wild sheep are steep mountainous woods near tree lines, and they are known to migrate to lower altitudes in winter. Today, the wild bezoar goat *Capra aegagrus* and the smaller wild urial sheep *O. orientalis* are only found in Afghanistan and northern Pakistan further south. Close species as the Siberian ibex *Capra ibex siberica* and the wild argali sheep *Ovis ammon* show present ranges extending into the Hindu Kush.

The possibility for wild boars *Sus scrofa* to have played an intermediate evolutionary role in this capture phenomenon may not be ruled out. Indeed, Suidae are evolutionarily related to hippopotamids (78) and the wild boar is an animal living both in warmer lowlands and cooler highlands (115). *Sus scrofa* was present in the Asian Near East (116–118). Moreover, the wild boar in western Europe (119) and feral black pigs in Sicily (120) are known to be infected by *F. hepatica*. Additionally, the pig has recently demonstrated to play an important role of reservoir for *F. hepatica*, at least in South America (121).

In this Asian Near East region, initial domestication steps with goats and sheep occurred around 11,000 years BP, and pigs and cattle domestication in that area started around 10,500 and 10,000 years BP, respectively. These dating estimations were 9,000–8,500 years BP in Mesopotamia, 10,000–8,500 years BP in Anatolia, and 9,600-8,500 years BP in the Levant (108).

Three archeo-parasitological findings of *F. hepatica* eggs are worth mentioning.

(A) Five *F. hepatica* eggs were found in sepultures on the island of Cyprus, dated back to 8,000–7,000 years BC (122, 123). These findings are the oldest archeological record of *F. hepatica*, and indicate that it was infecting domesticated animals at the earliest beginning of the domestication process. Human migration from the mainland into Cyprus took place between 11,100 and 10,600 years BP and goat, sheep, cattle, and pig were introduced into the island a bit later, around 10,400 years BP (124). *Fasciola hepatica* was able to maintain its life cycle thanks to the past presence of *G. truncatula* on the island, dated back around 7,200–6,800 years BC (125), which indicates that this lymnaeid vector was present in the neighboring mainland of Near East Asia in the past. This lymnaeid continues to be present in Cyprus until today (126).
(B) Four *F. hepatica* eggs were detected in a latrine of an archeological site in Israel dated back 200–300 years AC (122). This finding speaks about the past presence of this fasciolid in the Levantine corridor.
(C) The first archeological report of *F. hepatica* eggs in whole Asia concerns the finding in coprolites from a donkey in an archaeological site at an altitude of 1,663 m a.s.l. in the Zanjan province, northwestern Iran, dated back to the Sassanid period, 224–651 AD. This donkey was the Persian onager *Equus hemionus onager*, the Asiatic Wild-ass native of Iran. This finding indicates that this fasciolid had already spread through the Zagros mountains eastward from the Fertile Crescent at that time. Onagers were present in that region and neighborhood of the Fertile Crescent long before the beginning of the domestication of the livestock reservoirs of *Fasciola* (127).

In the Iranian province of Guilan, neighboring Zanjan province, surveys on goats, sheep, cattle and buffaloes showed a relationship of liver fluke infection with altitude, with *F. gigantica* transmitted by *R. auricularia* in the lowlands besides the Caspian Sea and *F. hepatica* transmitted by *G. truncatula* in the highlands of the Talesh mountains (128). In this zone, vertical transhumance pastoralism was practiced since 6,500 years ago (129). Vertical altitudinal transhumance and horizontal transhumance nomadic pastoralism should have significantly contributed to the dispersal of both *F. hepatica* and *F. gigantica* in domestic ruminants and their respective lymnaeid vector species during the Neolithic. Additional to the ruminants, equines and camelids should also have contributed in such an expansion process in the Near East, as suggested by their infection in present times (130, 131).

From this paleobiogeographical Asian Near East region of origin (Figure 6), *F. hepatica* was able to spread westward into Europe, eastward into Asia and southward into Africa by means of human-guided movements of domestic ruminants, equines and camelids along the post-domestication period, and subsequently to the Americas and Oceania after transoceanic ship transport in the last 500 years (1).

### Early introduction of *Fasciola gigantica* into Asia

*Fasciola gigantica* spread not only throughout its African continent of origin, but also Asia. Consequently, the question is posed on how, where and when was this fasciolid able to migrate out of Africa and reach the Asian continent, establish, and later expand up to the Far East, South East Asia and Pacific islands (1).

Paleontological data indicate that the eastern Mediterranean Levantine Corridor was the major route out for the African fauna into Eurasia, playing as a kind of bottleneck passage. This corridor is assumed to have been a northward extension of the East African Rift, with ecological and climatic characteristics similar to those of eastern Africa along the Plio-Pleistocene period. A high number of large mammal fossils of African origin have been reported from this Levantine Corridor (132). This corridor appears to have been the same route used by hippopotamuses and *R. natalensis* snails in their way out from the African continent.

This Levantine Corridor was, however, not always open. A land bridge connected Africa with Eurasia already in the Miocene according to the dramatic lowering of the level of the Mediterranean Sea. The fossil bovid record provides evidence for greater biological continuity between Africa and Eurasia in the late Miocene and earliest Pliocene than is found later in time (133). Although a great number of African faunal elements entered Eurasia around 18–17 mya (134), this was to early as *F. gigantica* was not yet originated. This bridge disappeared with the developing of the Red Sea and the flooding of the Mediterranean at the Miocene end, around 5 mya. During the subsequent Pliocene period of 5.33–2.58 mya, the barrier was maintained and the southern Levant was increasingly isolated. The quasi-isolation of this region finished with another regression of the Mediterranean toward the early Pleistocene (135). Other important faunal changes also occurred (i) at the beginning of the Pliocene around 5.5 mya, (ii) at the beginning of the Pleistocene around 2.0–1.8 mya, and (iii) at the early-middle Pleistocene boundary around 1.0 mya (134).

Among the African large mammals which used this corridor, there was a big assemblage of African bovids. The wide range of African animals indicates that there was no ecological selection on animals entering southwestern Asia (136). Three groups of these African bovids should be highlighted because of (i) being grazers, (ii) their ecological specializations linked to the presence of freshwater habitats, and (iii) showing the highest infection rates by *F. gigantica* in the wild fauna in Africa (Table 8), several even in the absence of or very rare contact with livestock. Indeed, bovid tribes and genera manifest evident relationships with given biome types which have been interpreted to underlie their original differentiation occurred along Miocene and Pliocene times (137):

(A) Reduncines are animals with a strong preference for flood plains and other areas close to water.
(B) Bovines of the cattle group live in floodplain grasslands and valleys within a short distance of water.
(C) Alcelaphalines live concentrated on greenbelts near water points in the dry season and disperse into dry savanna during the rainy season, preferring an open landscape with short grass, and they can also tolerate tree cover.

Among reduncines, the Plio-Pleistocene members of Asia belong to the extinct genus *Sivacobus* with evident relationships with the African crown kob cradle. *Sivacobus* is considered to have arisen from the same African ancestor of the kobs, which emigrated from Africa around 3.5 mya (138). Another fossilized species *Dorcadoxa porrecticornis* from the Siwaliks dated back 9.5–6.5 mya has also been considered to belong to the *Kobus* group because of its African relative *D. aff. porrecticornis*, which would mean an earlier entry of this African bovid group into Eurasia around the Late Miocene.

Among the bovines, the large buffalo *Bos* (*Pelorovis*) *oldowayensis* is considered the most significant element. The large buffalo *Pelorovis sensu stricto* is known to have been a common species in the African savannas in the Late Pliocene and Early Pleistocene, and has even been suggested to be the direct ancestor of the Middle Pleistocene Eurasian species *Bos primigenius* (132).

Among alcelaphalines, members of the genus *Damalops*, already extinct in Africa, have been found in Tajikistan dated to the Late Pliocene and were also present in the Siwaliks around 2.5 mya. Indeterminate alcelaphines were also recorded in southwestern Asia from the early Pleistocene onwards (136).

Hippotragines (in the Early to Middle Pleistocene) (136) and giraffids (in the Late Miocene and again later in the early Pleistocene) (134) were also included among the African immigrants, and may have also participated in the dispersal of *F. gigantica* once already in Asia, as they have been verified to be infected by this fasciolid in Africa (Table 8), although only secondarily given their less appropriate ecological preferences regarding the fluke life cycle.

It should, moreover, be considered that, additional to the aforementioned introduction of *R. natalensis*, polymorphic fossilized shells of *R. auricularia* indicate that this lymnaeid was widely present in the plain of Lower Mesopotamia during the Quaternary (139). *Radix auricularia* is currently found as a pronouncedly aquatic snail in permanent, still or slow flowing, and temporary collections of water of this plain (140), as well as in central Irak (141). Consequently, lymnaeid vectors able to assure the life cycle of *F. gigantica* were there.

Summing up, *F. gigantica* had many opportunities to emigrate from Africa into southwestern Asia and the possibility of more than an old introduction wave may even not be ruled out. All this indicates that *F. gigantica* should have been there transmitted by African-introduced *R. natalensis* and Asian-autochthonous *R. auricularia* in the lowlands of the Levant and Near East Asia at the time of the beginning of the animal domestication in the Fertile Crescent around 12,000–10,000 years ago (107–109). This should have allowed *F. gigantica* to benefit from herding and pastoralism for its spread throughout the Near East during the Neolithic and for its subsequent further expansion eastward into Asia by means of human-guided movements of the domestic herbivores including ruminants, equines and Old World camelids (1).

## Conclusions

The sequences of the nuclear rDNA and mtDNA markers show evidence of a close relationship of *F. gigantica* and *F. hepatica* of worldwide distribution with *F. nyanzae* of sub-Saharan Africa. The direct evolutionary derivation of the two former from the latter species agrees with the strict specificity of *F. nyanzae* in hippopotamus (older origin leading to long-term coevolution specialization) and the pronouncedly low specificity of the two other fasciolids in herbivore mammals, including a few omnivores like pigs and humans (modern origin only enabling a short-term coevolution insufficient for specialization). Fasciolid adult stage size show a length cline, from the biggest *F. nyanzae* to the smallest *F. hepatica*, manifesting a parallelism with the size of their main definitive hosts, which agrees with the recognized influence of the biliary canal inner space on fasciolid fluke size. Molecular clock dating estimates define a chronological scenario which adequately fits the fossil picture and paleobiogeographical events in Africa and Asian Near East.

The multidisciplinary analysis for *F. gigantica* suggests southeastern Africa as its paleobiogeographical region of origin, with a core zone around present-day Uganda and neighboring countries. Its chronological period for appearance should have been the mid-Miocene, around 13.5 mya. The original host capture phenomenon leading to speciation should have involved from the primitive hippopotamuses to bovid ancestors of Reduncinae, Bovinae, and Alcelaphinae, characterized by grazing preferences in flooded plains and proximity to freshwater collections. African *Radix natalensis* was the original lymnaeid snail vector species, and warm lowlands surrounding peaceful freshwater collections inhabited by hippopotamuses should have been the original biome (Figure 6).

For *F. hepatica*, all data suggest the Near East of Asia to have been its paleobiogeographical region of origin, from the Levant and Mesopotamia to the mountains of the Taurus, the Caucasus and Zagros. This fasciolid should have appeared after a host capture phenomenon from primitive, less amphibious *Hexaprotodon* hippopotamuses to southwest Asian ovicaprines (Bovidae: Caprinae) in the period of the latest Miocene to Early Pliocene, around 6.0–4.0 mya and perhaps shortly afterwards. The wild bezoar *Capra aegagrus* and the wild mouflon *Ovis gmelini*, direct ancestors of the domesticated goat *Capra hircus* and the domesticated sheep *Ovis aries*, respectively, should be considered the most probable original definitive hosts of *F. hepatica*, although a possible evolutionary intermediate passage through the wild boar *Sus scrofa* should not be ruled out. The lymnaeid *Galba truncatula* may be considered its original snail vector species after another freshwater snail capture phenomenon from radicine lymnaeids. The original ecological habitat should have included cooler areas and mountainous foothills, where the aforementioned hippopotamuses and mid-sized mammals overlapped (Figure 6).

## Data availability statement

The datasets presented in this study can be found in online repositories. The names of the repository/repositories and accession number(s) can be found in the article.

## Ethics statement

The animal study was reviewed and approved by the Evaluation of Projects concerning Animal Research at University of Valencia (Organo Habilitado para la Evaluación de Proyectos de Experimentación Animal de la Universidad de Valencia) (A1263 915389140), strictly following the institution's guidelines based on Directive 2010/63/EU. Permission for animal research was additionally obtained from the Servicio de Sanidad y Bienestar Animal, Dirección General de Producción Agraria y Ganadería, Consellería de Presidencia y Agricultura, Pesca, Alimentación y Agua, Generalitat Valenciana, Valencia, Spain (No. 2015/VSC/PEA/00001 tipo 2). Animal ethics guidelines regarding animal care were strictly adhered to Parasitological studies on hippopotamuses of Private Nature Reserve 1, Hoedspruit, the Private Nature Reserve 3 (New camp dam), and the Private Nature Reserve 2 of the Mpumalanga Province of South Africa were performed within an official permission agreement (Agreement document No. 13582). Written informed consent was obtained from the owners for the participation of their animals in this study. In Sri Lanka, liver flukes were obtained by dissection of naturally deceased wild elephants in fieldwork collaboration with members of the Minneriya National Park and the Department of Veterinary Pathobiology, Faculty of Veterinary Medicine and Animal Science, University of Peradeniya, under the auspices of the national Department of Wildlife Conservation (DWC). No ethics approval nor consent was needed for snail collections done on public land.

## Author contributions

MDB, AH, PA, WJL-P, MAV, and SM-C participated in the investigation. MDB, AH, WJL-P, and SM-C obtained the materials. AH and WJL-P coordinated local activities in South Africa. MDB, PA, MAV, and SM-C applied the methods. SM-C designed the study. MDB, WJL-P, and SM-C obtained and administered the project fundings. SM-C and MDB analyzed the results and wrote the manuscript. All authors read and approved the final manuscript.

## Funding

Field work on hippopotamus carcasses in South Africa funded by Project No. 101054 of the South African Research Chairs Initiative (SARChI) of the Department of Science and Innovation and the National Research Foundation of South Africa. Field work for the obtaining of samples in other countries and laboratory research in Spain funded by CIBER de Enfermedades Infecciosas Project No. CB21/13/00056, CIBER - Consorcio Centro de Investigación Biomédica en Red (CB 2021), Instituto de Salud Carlos III, Ministerio de Ciencia e Innovación, Madrid, Spain, and Unión Europea—NextGenerationEU; by the Red de Investigación de Centros de Enfermedades Tropicales—RICET (Project No. RD16/0027/0023 of the PN de I+D+I, ISCIII-Subdirección General de Redes y Centros de Investigación Cooperativa RETICS), Ministry of Health and Consumption, Madrid; by Projects Nos. 2016/099 and 2021/004 of the PROMETEO Program, Programa of Ayudas para Grupos de Investigación de Excelencia, Generalitat Valenciana, Valencia, Spain; and by Health Research Project No. PI16/00520, Subprograma Estatal de Generación de Conocimiento de la Acción Estratégica en Salud (AES) y Fondos FEDER, Plan Estatal de Investigación Científica y Técnica y de Innovación, ISCIII-MINECO, Madrid, Spain. The funders had no role in the design of the study; in the collection, analyses, or interpretation of data; in the writing of the manuscript; or in the decision to publish the results.

## Conflict of interest

The authors declare that the research was conducted in the absence of any commercial or financial relationships that could be construed as a potential conflict of interest.

## Publisher's note

All claims expressed in this article are solely those of the authors and do not necessarily represent those of their affiliated organizations, or those of the publisher, the editors and the reviewers. Any product that may be evaluated in this article, or claim that may be made by its manufacturer, is not guaranteed or endorsed by the publisher.
